# Fruit and Vegetable Supplemented Diet Modulates the Pig Transcriptome and Microbiome after a Two-Week Feeding Intervention

**DOI:** 10.3390/nu13124350

**Published:** 2021-12-02

**Authors:** Gloria I. Solano-Aguilar, Sukla Lakshman, Jonathan Shao, Celine Chen, Ethiopia Beshah, Harry D. Dawson, Bryan Vinyard, Steven G. Schroeder, Saebyeol Jang, Aleksey Molokin, Joseph F. Urban

**Affiliations:** 1 U.S. Department of Agriculture, Northeast Area, Agricultural Research Service, Beltsville Human Nutrition Research Center, Diet Genomics and Immunology Laboratory, Beltsville, MD 20705, USA; Sukla.Lakshman@usda.gov (S.L.); Celine.Chen@usda.gov (C.C.); Ethiopia.Beshah@usda.gov (E.B.); Harry.Dawson@usda.gov (H.D.D.); Saebyeol.Jang@gmail.com (S.J.); Aleksey.Molokin@usda.gov (A.M.); Joe.Urban@usda.gov (J.F.U.J.); 2 Statistics and Bioinformatics Group, Agricultural Research Service, U.S. Department of Agriculture, Northeast Area, Beltsville, MD 20705, USA; Jonathan.Shao@usda.gov (J.S.); Bryan.Vinyard@usda.gov (B.V.); 3 U.S. Department of Agriculture, Northeast Area, Agricultural Research Service, Beltsville Agricultural Research Center, Animal Genomics and Improvement Laboratory, Beltsville, MD 20705, USA; Steven.Schroeder@usda.gov

**Keywords:** fruit and vegetable, microbiome, immune response, 16S rDNA, RNA sequencing, WBC transcriptome

## Abstract

A study was conducted to determine the effects of a diet supplemented with fruits and vegetables (FV) on the host whole blood cell (WBC) transcriptome and the composition and function of the intestinal microbiome. Nine six-week-old pigs were fed a pig grower diet alone or supplemented with lyophilized FV equivalent to half the daily recommended amount prescribed for humans by the Dietary Guideline for Americans (DGA) for two weeks. Host transcriptome changes in the WBC were evaluated by RNA sequencing. Isolated DNA from the fecal microbiome was used for 16S rDNA taxonomic analysis and prediction of metabolomic function. Feeding an FV-supplemented diet to pigs induced differential expression of several genes associated with an increase in B-cell development and differentiation and the regulation of cellular movement, inflammatory response, and cell-to-cell signaling. Linear discriminant analysis effect size (LEfSe) in fecal microbiome samples showed differential increases in genera from *Lachnospiraceae* and *Ruminococcaceae* families within the order Clostridiales and *Erysipelotrichaceae* family with a predicted reduction in rgpE-glucosyltransferase protein associated with lipopolysaccharide biosynthesis in pigs fed the FV-supplemented diet. These results suggest that feeding an FV-supplemented diet for two weeks modulated markers of cellular inflammatory and immune function in the WBC transcriptome and the composition of the intestinal microbiome by increasing the abundance of bacterial taxa that have been associated with improved intestinal health.

## 1. Introduction

Short-term feeding of diets composed largely of animal-based or plant-based products to humans can rapidly shift the intestinal microbial composition and function [1]. A recent study of >1000 subjects showed that feeding a diverse healthy and plant-based diet to humans stratified the intestinal microbiome into organisms associated with markers of improved cardiovascular and postprandial glucose metabolic function [2]. Two independent cross-sectional data analyses of whole diets fed to American and Finnish populations, parsing out consumption of FV, showed an association between higher gut bacterial diversity and favorable microbiome composition with increasing systemic levels of total carotenoids and a healthy food choice index, respectively, supporting evidence that enhanced FV consumption improved host health through changes in the intestinal microbiome [3,4]. Further evidence has also linked the impact of FV consumption on host–microbiome composition and function, suggesting the possibility that certain microbial groups that are modulated by diet can affect human health [5,6,7]. A higher intake of FV or derived polyphenols has been associated with improved inflammatory and immune status in subjects with metabolic diseases [8,9,10,11], but further research is needed to elucidate the mechanisms of immunomodulation. Nutrigenomic studies have shown the impact of healthy diets on improved metabolic function and redox-associated genes that promote human health [12,13,14]. However, little is known about the impact of an FV-supplemented diet on the blood transcriptome and microbiome of healthy subjects. Therefore, using the pig as a translational model for testing nutritional interventions, we evaluated the relationship between an altered intestinal microbiome induced by feeding an FV-supplemented diet on changes in gene expression in WBC for markers of improved health. The goal of this study was to evaluate the effect of a two-week intervention diet of FV consumption based on the Dietary Guidelines for Americans (DGA) on the pig WBC transcriptome and fecal microbiome as a translational model for similar studies in humans.

## 2. Materials and Methods

### 2.1. Animals and Diets

All animal experiments and procedures were conducted in accordance with the guidelines established and approved by Beltsville Area Animal Care and Use Committee under protocol 19-016. Fresh fruits (grapes, strawberries, red apples, blackberries, and blueberries) and vegetables (celery, broccoli, spinach, and kale) were purchased from local markets and cut and separately weighed in labeled cups before lyophilization. A daily volume of 1.25 cups of fruit and 1.75 cups of vegetables were measured to provide approximately 50% of the DGA recommendation for a healthy-style diet consumed by adults between 19 to 59 years of age with an intake of 2600 kilocalories per day (www.dietaryguidelines.gov, accessed on 26 November 2021). In addition, two servings of 4.5 ounces of chicken breast were also lyophilized and included in the FV-supplemented diet to provide approximately 6% of the recommended daily level of protein and fat from an animal source generally consumed by humans. The nutrient composition of the lyophilized chicken and FV mixtures was analyzed by Eurofins (Eurofins Scientific Inc., Des Moines, IA, USA) and used for diet calculations to provide a similar calorie content to the control-non supplemented diet (Table S1). Nine Large White X Landrace six-week-old pigs born within the same week were obtained from two litters weaned and individually housed in pens in a single nursery barn located at the USDA Swine facility in Beltsville, MD. Pigs were randomized by weight and gender into two experimental treatment groups at week 6. Group I (*n* = 4, 2 females, 2 males) was fed a regular pig grower diet with 17% energy (E) from fat, 62% E from carbohydrates, and 20% E from protein and designated as the control group. Group II (*n* = 5, 3 females, 2 males) was fed a similar grower diet supplemented with FV and chicken. Both diets contributed similar calories from macronutrients but with differences in carbohydrate, protein, and fiber sources. Pigs were fed ad libitum, and feeder contents were monitored daily for consumption of expected amount of diet. Pigs were weighed, and blood and feces were collected at the start and end of the two-week dietary intervention. All pigs were euthanized by IV injection with Euthasol (50 mg sodium pentobarbital/kg of body weight) (Virbac Animal Health, Inc., Fort Worth, TX, USA) at the end of the study. 

### 2.2. Transcriptome Response to Dietary Intervention

Whole blood cells (WBC) were collected in Paxgene collection tubes (BD Biosciences, Franklin Lakes, NJ, USA) and processed for RNA extraction, as previously described [15,16]. Illumina TruSeq RNA Sample Prep v2 kits (Ilumina, San Diego, CA, USA) were used to prepare RNA sequencing libraries from WBC according to manufacturer protocol. The combined library pool was denatured and loaded on a Illumina NextSeq 500 sequencer (Illumina, San Diego, CA, USA), using a high output flow-cell to generate 150 base pair single-end reads. Base-call conversion, de-multiplexing, and adapter trimming of the pooled sequence data were performed using bcl2fastq2 conversion software (v2.20.0.422, Illumina, Inc.). Unaligned FASTQ files generated from sequencing were imported into CLC Genomics Workbench version 11.0 (QIAGEN, Bioinformatics, Redwood City, CA, USA), where they were trimmed to remove low-quality reads and adapter sequences. Reads were first mapped to a custom, non-redundant (NR) (NR_111919v2) 8959 transcript library, as previously described [17,18]. The NR library contains the sequences of genes involved in immunity/inflammation and genes involved in macro and micronutrient metabolism. The sequences for these genes are found in the Porcine Translational Research Database maintained by the Beltsville Human Nutrition Research Center, Diet, Genomics, and Immunology Laboratory (http://tinyurl.com/hxxq3ur, accessed on 26 November 2021) [19]. Inclusion of the NR library is necessary because Ensembl build 11.1 contains a large number of documented errors [20,21], and the 5′ and 3′ untranslated regions of genes tend to be underrepresented by the Ensembl algorithm [19]. Unmapped reads were then mapped to the Ensembl build 11.1 v98 build 11.1 (WG) to account for expressed genes that were not covered by the NR library. Mapped reads for each sample derived from both reference libraries were combined into gene-level expression counts that were used as input for differential gene expression (DGE) analysis. RNA isolated from WBC were converted to cDNA via the iScript cDNA synthesis Kit (Biorad, Hercules, CA, USA). Briefly, 25 ng/well of cDNA in duplicates from each biological sample were used for real-time PCR amplification using iTaq Universal Probes Supermix (Biorad, Hercules, CA, USA) and the ABI PRISM 7500 Sequence detector system (Applied Biosystems, Foster City, CA, USA). A subset of specific porcine primers and probe sequences against selected inflammatory or immune response-related genes were synthesized by Biosearch Technologies (Novato, CA, USA), as described in the Porcine Translational Research Database [19] and used for validation of DGE associated with dietary treatment after RNA-seq analysis. Gene expression was normalized to the housekeeping gene RPL32 using the 2^−ΔΔCT^ method [22] and expressed as a fold change compared to baseline or control treatment group. The fold change was calculated using the mean difference of the treatment group, as previously described [23].

### 2.3. Fecal Specimen Collection and Processing for 16S rDNA Amplicon Multi-Tag Sequencing and Metabolic Prediction

Fresh fecal samples were directly collected from the rectum of pigs using a cotton swab to stimulate defecation. A 5 g aliquot of each fecal sample was collected in a sterile 50 mL plastic tube before starting the dietary intervention at day 0 (baseline) and two weeks later. One-gram aliquots were immediately weighed and stored at −80 °C until further processing. DNA was extracted using QIAamp DNA stool kit (Qiagen, Germantown, MD, USA) with an initial disruption with ceramic beads (Precellys, Krackeler Scientific, NY, USA) using two 30-s disruption cycles with 5000 rpm and a 95 °C heat step [15]. DNA samples were cleaned and concentrated with ZR Genomic DNA Clean and Concentrator-25 (Zymo Research, Irvine, CA, USA). DNA was quantified with Quantt-iT dsDNA Assay Kit (Invitrogen, Carlsbad, CA, USA) using a Spectra-Max multimode microplate reader (Molecular Devices, San Jose, CA, USA) and DNA quality determined by Nanodrop (Thermo Fisher Scientific, Wilmington, DE, USA). 16S rRNA libraries were prepared using the V3-V4 hypervariable regions of 16S rRNA, as described in 16S metagenomic sequencing library preparation manual (https://www.illumina.com/search.html?filter=support&q=16S%20metagenomic%20sequencing%20library&p=1, accessed on 26 November 2021). The quality and size of the libraries were verified using the Agilent DNA 1000 kit and run on the Agilent 2100 Bioanalyzer (Agilent Technologies, Santa Clara, CA, USA). Library quantification was completed using the KAPA Library Quantification kit (KAPA Biosystems, Wilmington, MA, USA). Libraries were normalized to 4 nM before pooling equal volumes. The final library concentration was 6 pM with PhiX control v3 (15%, *v/v*) (Illumina San Diego, CA, USA). Libraries were sequenced using Illumina Miseq sequencer and a 600-cycle Miseq Reagent kit v3. Sequences were demultiplexed using the dual-index strategy, the mapping file generated on the robotic platform, and split_libraries_fastq.py, a QIIME-dependent script [24]. All sequences were dereplicated and the resulting forward and reverse fastq files were split sample using seqtk (https://github.com/lh3/seqtk, accessed on 26 November 2021), and primer sequences were removed using TagCleaner (0.16) [25]. Paired-end reads were further cleaned and scanned for adaptors with bbduk from BBTools software suite. The paired-end reads were merged with bbmerge from the BBtools software suite to verify that the paired-end reads overlapped (https://jgi.doe.gov/data-and-tools/bbtools/, accessed on 26 November 2021). Cleaned paired-end sequences were imported into Quantitative Insights into Microbial Ecology, QIIME2 (qiime2-2018-4) (https://qiime2.org, accessed on 26 November 2021), and the quality of sequences was visualized using a demux object. The first 9 nucleotides for each paired read were trimmed and the total length of reads was truncated to 250 base pairs for the forward and reverse reads to remove low-quality bases at the end of the reads. Filtered reads were input into DADA2, a denoising pipeline incorporated in QIIME2, to remove chimeric variants and to identify amplicon sequence variants (ASV) which have been shown to have a better taxonomic resolution [26]. Non-chimeric sequences were used for taxonomic classification of marker gene sequences using the q2-feature-classifier [27] with Silva v138 for all taxonomic identification and prediction of bacterial metabolic functions using a pairwise identity threshold of 97%. ASV-16S rRNA gene sequencing data were also used to generate metagenome predictions for Enzyme Commission number (EC number), relative Kyoto Encyclopedia of Genes and Genomes (KEGG) orthologs (KO), or metabolic pathways using the Phylogenetic Investigation of Communities by Reconstruction of Unobserved States (PICRUSt2) [28]. Pathways were calculated based on the abundance of gene families linked to reactions within pathways based on predicted EC numbers regrouped to MetaCyc reactions [29].

### 2.4. Statistical Analysis

Whole blood cell gene counts derived from pigs in each dietary treatment group were used to identify DGE. Sequences below quality score (Q30)dsaXZ or reads containing more than two ambiguous nucleotides were removed before sequence alignments were performed using the CLC Genomics Workbench version 12 (Qiagen Bioinformatics, Redwood City, CA, USA). Gene expression was normalized using the “reads per kilobase of exon model per million mapped reads” model (RPKM) [30]. Only genes with RPKM values above 1.5 were included in the downstream analysis. Differentially expressed gene lists obtained from the WG and NR libraries were compared with an online two lists tool (http://barc.wi.mit.edu/tools/compare, accessed on 26 November 2021). Principle component analysis (PCA) and hierarchical clustering were performed with JMP Genomics 10 (SAS, Cary, NC, USA). The differential expression analyses were carried out with the Bioconductor package DeSeq2 v 3.14 [31] as recommended for datasets with low number of replicates [32]. Genes were considered differentially expressed with the threshold of a false discovery rate (FDR) ≤0.05 and an absolute fold change ≥1.5. Biological network analysis was performed using Ingenuity Pathway Analysis (IPA) (v9.0 Ingenuity Systems, Mountain View, CA, USA) to predict potential biological processes, pathways and molecules affected by DGE. Networks of these focus genes were algorithmically generated based on their connectivity and the number of focus genes. To identify the networks that were highly expressed, IPA computes a score per the fit of the genes in the data set. This score was generated using a *p*-value calculation determined by a right-tailed Fisher’s exact test and indicates the likelihood that the fit of the focus genes in the network could be explained by chance alone. Z-score serves as both a significance measure and a predictor of the activation state of the gene: activated (Z value > 2) or inhibited (Z value < 2) [32,33]. The goal was to identify biological processes and functions that were likely to be casually affected by up- and downregulated genes from the data generated. To further interpret the biological meaning of DGE in WBC after dietary interventions, the overlap between our gene dataset was compared with previously deposited gene sets from the Molecular Signature database (MsigDB) [34] so that common genes with other datasets could be related.

Bioinformatic analysis of Amplicon Sequence Variants (ASV) in fecal samples was done using Quantitative Insights into Microbial Ecology, QIIME2 (qiime2-2018-4) [27,35]. After removal of low ASV counts (less than 0.01%), ASV counts were normalized by cumulative-sum scaling (CSS), and log2 transformation to account for the non-normal distribution of taxonomic counts data [36]. Alpha (within samples) diversity was calculated to reflect the influence of treatment on the structure of fecal microbiome through Shannon alpha diversity index at the genus level. Several tools such as Principal coordinates Analysis (PCoA), and Analysis of Similarities (ANOSIM) to compare the mean of ranked dissimilarities between groups to the mean of ranked dissimilarities within groups were used for beta diversity analysis based on Bray–Curtis distance matrix [37]. Multivariate analysis using Canonical Correspondence Analysis (CCA) was also implemented to assess if variations in the data matrix can be explained by grouping variables: diet, time, and their interactions in one coherent graphical model. The linear discriminant analysis effect size (LEfSe), an algorithm for biomarker discovery that identifies enrichment of abundant taxa or function between two or more groups, was used to compare all taxa at different taxonomic levels simultaneously (i.e., phylum, class, order, family, and genus). This method uses a linear discriminant analysis (LDA) model with continuous independent variables to predict one dependent variable and provides an effect size for the significantly different taxa or metabolic function based on relative differences between variability and discriminatory power [38]. Finally, ASV data table was imported into PICRUSt2 (Phylogenetic Investigation of Communities by Reconstruction of Unobserved States) and ran through the picrust2_pipeline.py script for sequence placement, hidden-state prediction of genomes, metagenome prediction, and pathway-level predictions (https://github.com/picrust/picrust2, accessed on 26 November 2021). After a center log-ratio transformation of the data, as previously described [39], an analysis of variance (ANOVA) was used for multiple-group statistical analysis with Tukey–Kramer post-hoc test to determine which means were significant, followed by Benjamini-Hochberg FDR for multiple test correction. Two group statistical analysis was also done with a two-sided Welch’s *t*-test with confidence intervals and Benjamini-Holchberg FDR multiple test correction using Statistical Analysis of Metagenomic Profiles (STAMP) software v2.1.3 to identify significant differences in relative abundance of predicted metagenomic pathways between dietary treatment groups [40].

## 3. Results

### 3.1. Clinical Signs 

All pigs gained weight over the two-week dietary intervention, with no differences in growth rate and no clinical signs of disease in response to dietary treatment (Figure S1). 

### 3.2. Fruit and Vegetable Supplemented Diet Effect on Blood Transcriptome 

**Figure 1:**
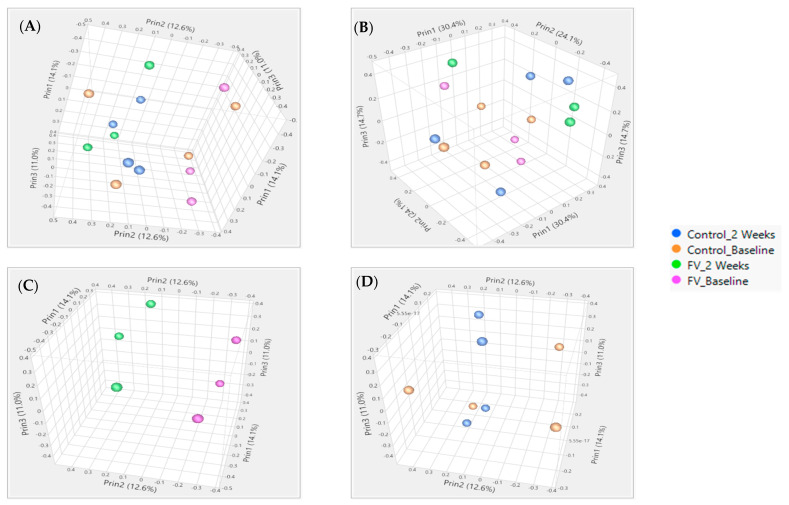
Principal component analysis of pigs fed a fruit and vegetable (FV)-supplemented or control diet (**C**) for two weeks. Separation of pigs with collection times within each diet as specified in the figure legend showed more clustering in FV dietary group than in control group with 14.1%, 12.6%, and 11.0% variation with the first three components for NR (**A**) and 29.0%, 16.5%, and 14.2% for WG (**B**) databases, respectively. PCOA with separate paired sample clustering by time in pigs from FV diet group (**C**). No clustering by time in pigs from control diet group (**D**).

RNA derived from paired WBC collections at baseline and two weeks post-intervention were processed for pigs from the control (*n* = 4) and FV (*n* = 3)-supplemented dietary groups as independent replicates for mapping reads [41]. Principal component analysis (PCA) using the NR or WG dataset showed the complete separation of pigs from the FV-supplemented dietary group and partial separation of pigs from the control group when paired samples collected at two weeks post-intervention and baseline were compared. No separation between dietary groups was visualized at baseline or two weeks post-intervention (Figure 1). 

**Figure 2:**
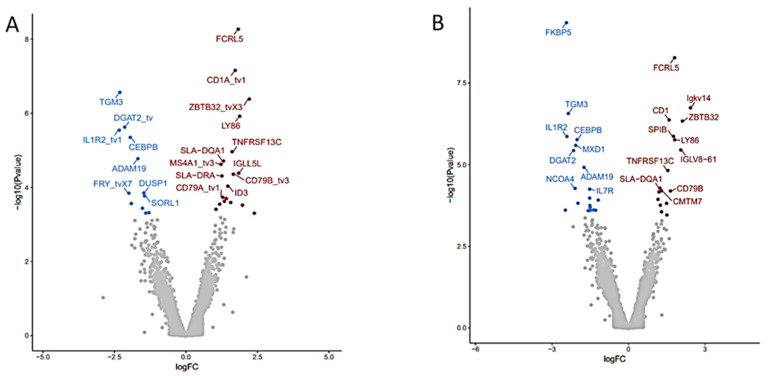
Differential gene expression (DGE) analysis of RNA-seq data derived from pigs fed FV-supplemented relative to control diet after two weeks. Volcano plots depicting the fold difference in gene expression levels after consumption of FV supplemented diet relative to controls after two weeks when genes were mapped with NR (**A**) or WG (**B**) reference libraries. An absolute threshold fold change of 1.5 with an adjusted FDR ≤ 0.05 was applied to capture significant gene expression differences (red for upregulated genes, blue for downregulated genes).

Differential gene expression analysis was determined using the DeSeq2 v 3.14 Bioconductor package. Volcano plots illustrating DGE in WBC of pigs fed the supplemented FV diet relative to the control diet illustrate a similar number, with some differences in function for DGE depending on the reference library used with 31 DGE in NR (19 upregulated, 12 downregulated) (Figure 2A) or 37 DGE in WG (18 upregulated, 19 downregulated) databases (Figure 2B) (Table S2). 

**Figure 3:**
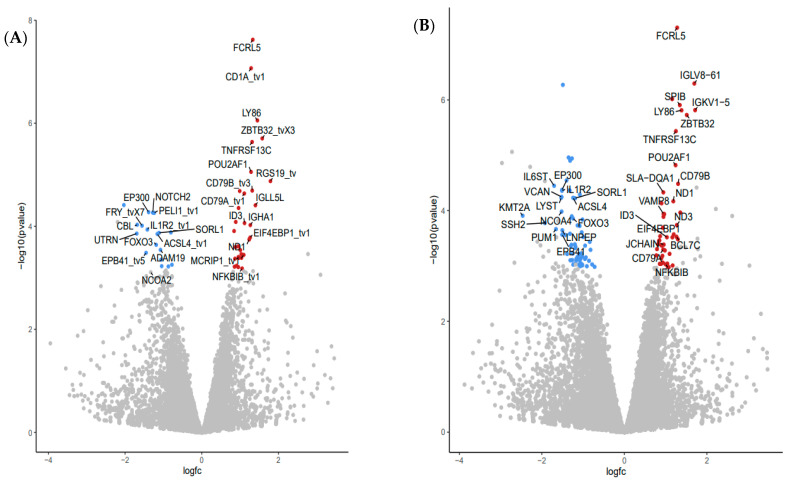
Differential gene expression (DGE) analysis of RNA-seq data derived from pigs fed FV-supplemented diet after two weeks. Volcano plots depicting the fold difference in gene expression levels after consumption of FV supplemented diet relative to their paired-baseline sample after genes were mapped with NR (**A**) or WG (**B**) reference libraries. An absolute threshold fold change of 1.5 with an adjusted FDR ≤0.05 was applied to capture significant gene expression differences (red for upregulated genes, blue for downregulated genes).

Top common upregulated DGE found in two genome reference libraries (Table S2) included: transmembrane proteins coded by Fc receptor-like 5 (*FCRL5*), CD1a molecule (*CD1*), CD79B molecule (*CD79B*), lymphocyte antigen 86 (*LY86*), TNF receptor superfamily member 13C (*TNFRSF13C*); transcriptional regulator zinc finger and BTB domain containing 32 (*ZBTB32*), and downregulated DGE included plasma transmembrane molecule interleukin 1 receptor type 2 (*IL1R2*); plasma enzymes: ADAM metallopeptidase domain 19 (*ADAM19*), transglutaminase 3 (*TGM3*), diacylglycerol O-acyltransferase 2 (DGAT2) and transcriptional regulator: CCAAT enhancer-binding protein beta (*CEBPB*). Many more DGE were identified when annotated genes in WBC from pigs fed the FV supplemented diet were compared to their paired baseline sample taken at the start of the intervention. Volcano plots comparing the outputs with both genome mapping libraries indicated 49 DGE (31 upregulated, 18 downregulated) (Figure 3A) and 111 DGE (46 upregulated and 65 downregulated) (Figure 3B) when the NR and WG genome references were used, respectively. Among the top common upregulated DGE (Table S3), there were transcriptional regulators: POU class 2 homeobox associating factor 1 (*POU2AF1*), inhibitor of DNA binding 3 HLH protein (*ID3*), NFKB inhibitor beta (*NFKBIB*); plasma membrane: eukaryotic translation initiation factor 4E binding protein 1 (*EIF4EBP1*); enzyme: NADH dehydrogenase subunit 1 (*ND1*); plasma membrane molecules: CD79a (*CD79A*), immunoglobulin lambda-like polypeptide 5 (*IGLL5L*) in addition to DGEs also detected in WBC of pigs fed the FV-supplemented diet relative to controls (i.e., *FCRL5*, *CD1*, *LY86*, *TNFRSF13C*, and *ZBTB32*). Downregulated DGE included transcriptional regulators E1A binding protein p300 (*EP300*), notch receptor 2 (*NOTCH2*) plasma membrane: erythrocyte membrane protein band 4.1 (*EPB41*), enzymes: pellino E3 ubiquitin-protein ligase 1 (*PELI1*), acyl-CoA synthetase long-chain family member 4 (*ACSL4*) and transporter: sortilin related receptor 1 (*SORL1*) in addition to DGE also downregulated in WBC of pigs fed FV-supplemented diet relative to controls (*IL1R2*, *FOXO3*, *ADAM19*). Real-time PCR validation was confirmed for selected genes (Table S4). No DGE was found between paired samples from the control diet group. Four DGE non-related with immune function were not corrected by randomization in baseline samples among treatment groups (Table S5).

### 3.3. Biological Interpretation of Differential Gene Expression

**Figure 4:**
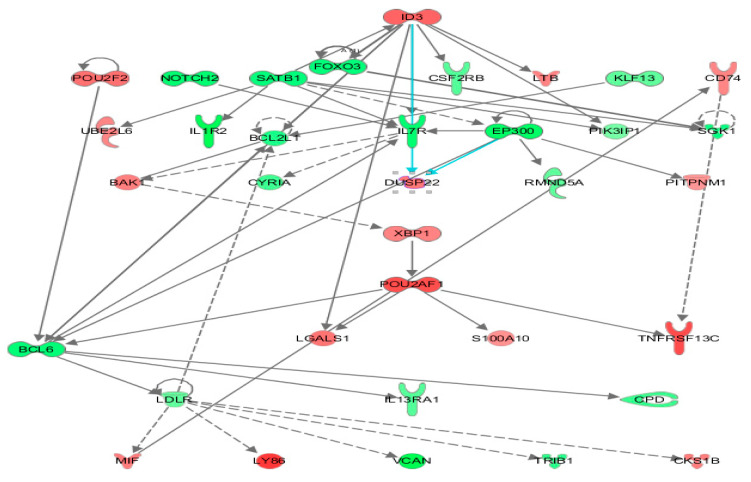
Ingenuity top gene network reflecting immune response-related transcriptome changes in whole blood from pigs fed an FV-supplemented diet. Nodes in the interaction network are encoded by DGE detected by DESeq2 in WBC from pigs after two-week consumption of an FV-supplemented diet. Upregulated genes are depicted in shades of red and downregulated genes in shades of green.

Differential gene expression derived from both reference libraries were combined into a single list of DGE and used as input for predicting the impact of the FV-supplemented diet on modulating WBC biological function after two weeks (Table S3). The Ingenuity pathway analysis (IPA) platform was used to identify the affected top biological networks. The highest associated network function, with a score of 56, identified by IPA corresponded to Hematological system development and Function, Tissue Morphology, Lymphoid Tissue Structure and Development with 35 affected focus molecules that included many transcriptional regulators and repressors (i.e., *ID3*, *FOXO3*, *NOTCH2*, *EP300*, *POU2AF1*, SATB homeobox 1 (*SATB1*), BCL6 transcription repressor (*BCL6*) (Figure 4) which induce downstream effects on expression of genes coding for transmembrane receptors: interleukin 7 receptor (*IL7R*) and interleukin 1 receptor type 2 (*IL1R2*), colony-stimulating factor 2 receptor subunit beta (*CSF2RB*), *TNFRSF13C*, interleukin 13 receptor subunit alpha 1 (*IL13RA1*), transporters: low-density lipoprotein receptor (*LDLR*), phosphatidylinositol transfer protein membrane-associated 1 (*PITPNM1*), cytokines: lymphotoxin beta (*LTB*), macrophage migration inhibitory factor (*MIF*) and lectins: galectin 1 (*LGALS1*) contributing to differentiate the transcriptome after FV dietary intervention. 

**Figure 5:**
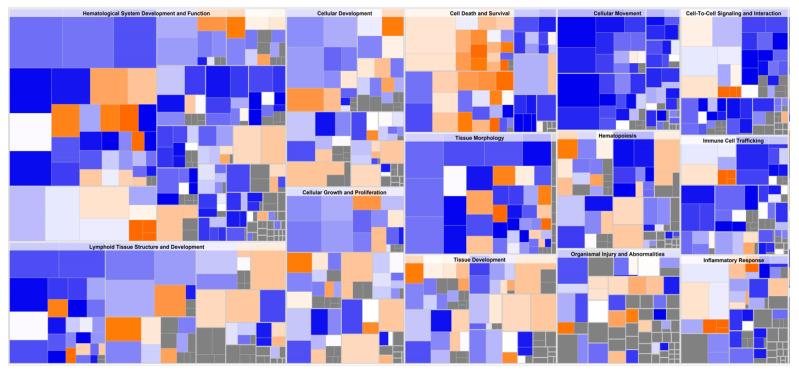
Downstream effect analysis on whole blood cells (WBC) from pigs fed an FV-supplemented diet. Hierarchical heatmap generated from DeSeq2 analysis, where the major boxes represent a category of related functions. Each individual colored rectangle is a particular biological function or disease, and the color indicates its predicted state: (increase: orange, decrease: blue). Darker colors indicate higher absolute Z scores. In this view, the size of the rectangle is correlated with increasing number of genes. The original image has been cropped for better readability of top affected functions. Specific functions with associated Z-score < 2.5 are summarized in Table S6.

These genes are known to exert a transcriptional control of several downstream effects affecting immune functions, including T-cell development, migration of follicular helper T-cells [42], shaping Th1/Th2 differentiation [43,44], regulation of dendritic cell activity [45], function as a coactivator of transcription factors that regulate immunoglobulin expression, and other host defense-related responses [46]. A heat map generated by the Diseases and Functions option tool within IPA illustrated the top biological categories (ranked by highest absolute Z-activation score and number of molecules) related to cellular movement, inflammatory response, cell-to-cell signaling, and hematological system development that were predicted to be casually affected by the transcriptomic changes encounter in WBC of pigs fed a supplemented FVdiet (Figure 5). Specific functions associated with migration of cells, immune cell response, binding of mononuclear cells, and lymphocytes and adhesion (Z-score < 2.5) were predicted to be downregulated (Table S6). To relate gene expression changes to previously described functional profiles, DGE in WBC of pigs fed FV-supplemented diet were overlapped with gene sets within the MSigDB database (https://www.gsea-msigdb.org/gsea/msigdb/, accessed on 26 November 2021) that represent cell states and perturbations within the immune system [34]. Our dataset showed overlap, with 10 out of 5219 data sets related to the induction of memory cell response and B-cell stimulation (Table S7).

### 3.4. Fruit and Vegetable-Supplemented Diet Affected Composition of Fecal Microbiome 

**Figure 6:**
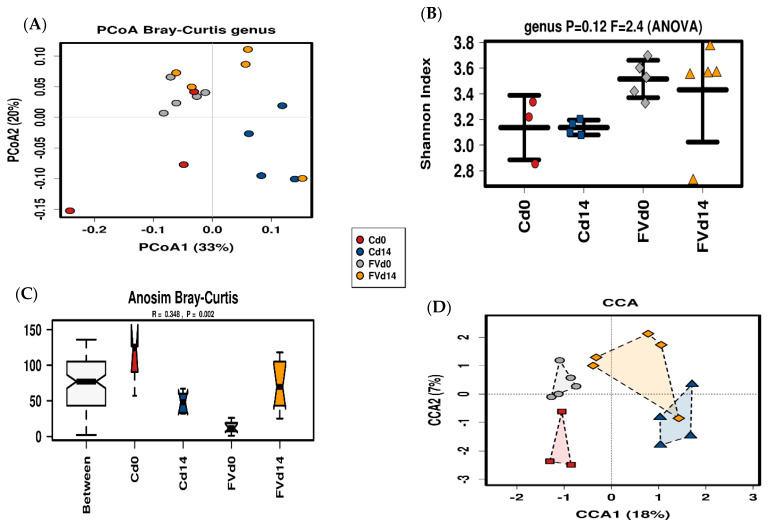
Fecal Microbiota (FM) Diversity. Principal coordinate analysis with each unique symbol described in the legend representing FM from pigs from the fruit and vegetables (FV) dietary group at day 0 (FVd0) and day 14 (FVd14) or control (**C**) diet groups at day 0 (Cd0) and day 14 (Cd14) (**A**). Bacterial alpha diversity (Shannon index) distribution is summarized with a line in the median (**B**). Analysis of similarity among treatment groups based on Bray-Curtis dissimilarity index (**C**) and supervised Canonical Correspondence Analysis (**D**) displaying the composition distribution of the FM for both diets at d0 and d14.

Sequencing of the V3-V4-region of bacterial 16S rRNA gene derived from samples of the pig fecal microbiome (FM) collected at the start (day 0) and two weeks after dietary intervention (day 14) with FV-supplemented diet or control diets produced a total of 1,245,477 reads after quality filtering and removal of one low-count sequence from baseline for a group sequence mean ± SD of 73,263 ± 38,994 reads per sample used for taxonomic analysis (*n* = 17) (Table S8). To compare FM composition among treatment groups, distance matrices were calculated by weighted unifrac and visualized using Principal coordinate analysis (PCoA) with 33% and 20% of the variation from the first two components (Figure 6A). No changes in alpha diversity were detected by Shannon diversity index between treatment groups (Figure 6B). Beta diversity (distance between FM groups) was significantly higher than the within-group distances (ANOSIM R value = 0.32, *p* = 0.009) (Figure 6C). FM data ordination by multivariate Canonical Correspondence Analysis (CCA) showed FM communities clustering by time (*p* < 0.05) but not diet (*p* = 0.19) indicating that a two-week intervention significantly affected FM composition independent of diet (Figure 6D) with no changes in alpha diversity as has been previously described in established FM of healthy pigs after weaning [47]. 

Among bacterial and Archaea phylum, Firmicutes (53.8%), Bacteroidetes (29.6%), Proteobacteria (6.1%), Actinobacteria (2.7%), Spirochaetes (0.9%), and Euryarchaeota (0.5%) were the most abundant phylum within the FM. At the family level, *Prevotellaceae* (23.9%), *Veillonellaceae* (13.8%), *Ruminococcaceae* (12.6%), *Lachnospiraceae* (12.0%), and *Lactobacillaceae* (9.5%) had the highest relative abundance with genus Prevotella 9 (12.2%), Lactobacillus (10.40%), Succinivibrio (6.2%), Megasphaera (4.7%), Subdoligranulum (3.0%) and genus *Ruminococcaceae UCG-014* group (2.5%) as the top dominant genus across all samples. 

**Figure 7:**
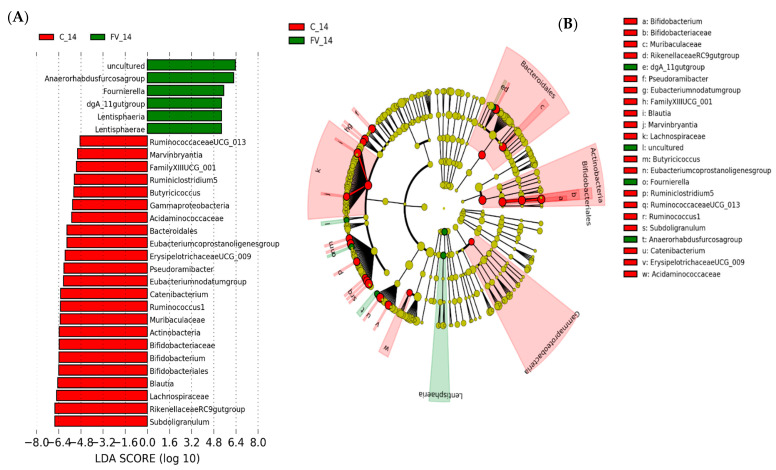

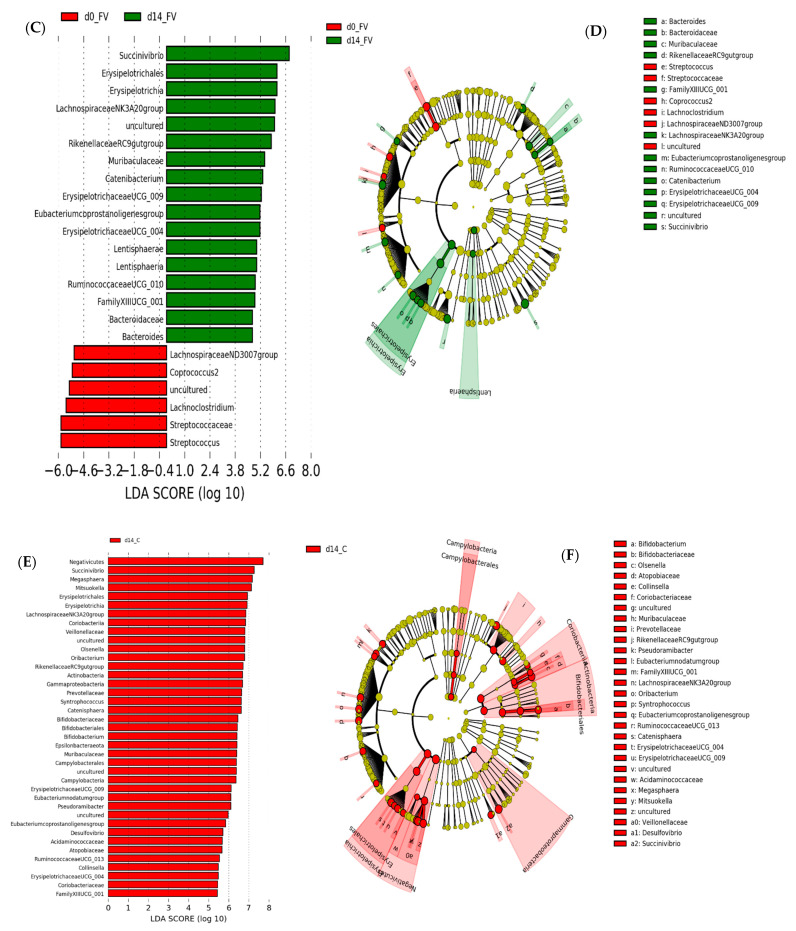
Linear discriminant analysis effect size (LEfSe) analysis representing differentially abundant taxa in pig fecal microbiome (FM). (**A**). Horizontal bars representing individual enriched ASVs in the FM of pigs fed fruit and vegetable supplemented (FV) or control (**C**) diet after 2 weeks. (**B**). Cladogram generated by the LEfSe method indicating phylogenetic distribution of FM associated with FV or C-fed pigs after two weeks integrating differential ASV at all taxonomy levels (**C**). Horizontal bars representing individual enriched ASVs in FM of pigs fed FV-supplemented diet at baseline (FV_0) or two weeks after intervention (FV_14) with (**D**). Cladograms integrating changes at all taxonomic levels in FV treated group. (**E**). Individual enriched ASVs in FM of pigs fed Control diet for 2 weeks with (**F**). Cladogram integrating changes at all taxonomic levels. Only taxa with linear discriminant analysis (LDA) scores *>* 2 are presented.

Differentially abundant taxa between dietary treatments were examined using LEfSe for biomarker (i.e., bacterial taxa) discovery applying effect size estimation. Relative to the FM of the control-diet group, FM from FV-supplemented pigs showed an increase in genus Fournierella from *Ruminococcaceae*, Anaerorhabdus fucosa group from *Erysipelotrichaceae,* and a single genus from the dgA_11 group within *Rikenellaceae* family with an increased abundance in ASVs within order Lentisphaeria and phylum Lentisphaerae (Figure 7A), while pigs in the control group maintained an increased abundance of *Muribaculaceae* and *Rikenellaceae* families within Order Bacteroidales, *Bifidobacteriaceae* within phylum Actinobacteria and several genera from *Lachnospiraceae* and *Ruminococcaceae* within Clostridiales (Figure 7B). Differential increase in genera within Class Erysipelotrichia (Erysipelotrichaceae spp., Catenibacterium) and Lentisphaeria was also detected in FV-supplemented pigs relative to their paired baseline sample (Figure 7C). In addition, there was also an increase in the abundance of several genera within order Bacteroidales, including ASVs from *Bacteroidaceae* (Bacteroides), *Muribaculaceae* and *Rikenellaceae* (Rickenellaceae RC9 gut group) families and order Clostridiales, including ASVs from *Ruminococcaceae* (*Ruminococcaceae* UCG010), *Eubacteriaceae* (*E. coprostanoligenes*), *Lachnospiraceae* (*Lachnospiraceae* NK3A20 group), and Family XIII (Figure 7C) while *Streptococcaceae* and some genera within *Lachnospiraceae* family were not affected by FV intervention Figure 7C,D. On the other hand, pigs maintained with the control diet showed an overall shift relative to baseline levels with increased abundance in *Bifidobacteriaceae*, *Atopobiaceae* and *Coriobacteriaceae* within Actinobacteria, *Prevotellaceae*, *Rikenellaceae* and *Muribaculaceae* within Bacteroidales, *Acidaminococcaceae,* and *Veillonellaceae* within Negativicutes and Erysipelotrichales concomitant with the diet induced dynamic shift post-weaning [47] and the appearance of some potential pathogens within phylum Gammaproteobacteria and Campylobacteria (Figure 7E,F). No changes at the phylum level were detected between assigned treatment groups at week 0; however, at a lower phylogenetic level, the order Clostridiales was more abundant (~2%) in pigs assigned to the FV-supplemented group, mainly driven by one pig with a higher abundance. Genus Collinsella (~1.5% relative abundance) and Christensenellacea R7 group (~7% relative abundance) also showed an increased abundance relative to the assigned control group, suggesting a bias in the abundance of these genera that was not corrected by the process of randomization (Figure S2). Predictive Functional Analysis with Phylogenetic Investigation of Communities by Reconstruction of Unobserved States (PICRUSt) was used to predict metagenomic functional content from the bacterial phylogenetic profiles observed using a curated collection [48]. The comparison of imputed relative abundances for KEGG metabolic pathways derived from FM bacterial taxa of FV and control diet-fed pigs showed a reduced abundance of K12998_rgpE; glucosyltransferase [E.C: 2.4.1.] and K01446_PGRP; peptidoglycan recognition protein in FV-fed pigs after a two-week feeding (FDR < 0.05) (Figure S3). 

## 4. Discussion

This study showed that daily consumption of a diet containing 1.25 cups of fruit and 1.75 cups of vegetables for two weeks positively affected the composition of the fecal microbiome (FM) of pigs by promoting the abundance of several genera within the *Erysipelotrichaceae* family, which have been previously correlated with dietary fiber consumption [49,50], adherence to the Mediterranean diet (MD) [51], higher systemic tocopherol concentrations as a marker of FV consumption in humans [3], and negatively correlated with inflammatory markers in weaned pigs [52]. Other genera from *Rikenellaceae* and *Muribaculaceae* families previously associated with complex metabolism of carbohydrates [53] and responsive to dietary polyphenolic compounds [54] were also increased in FV-supplemented pigs. The relative abundance of other major contributors to carbohydrate metabolism, such as genera within phylum Lentisphaerae, family *Ruminococcaceae* [55], and genus Bacteroides, was also increased in pigs fed the FV-supplemented diet relative to paired baseline samples, with fewer differentially abundant taxa when compared to pigs fed the control diet. Other environmental factors such as litter genetics, differences in FM at baseline, or dietary source of protein and fat used to balance the calorie content in the FV-supplemented diet may have contributed to enhanced response relative to its baseline level. However, the FV-induced abundance of health-promoting bacteria detected was likely due to more complex non-digestible plant fiber (also known as non-digestible polysaccharides) and polyphenols that are abundant in fruits and vegetables [56,57] and have compelling evidence from preclinical studies to modulate gut dysbiosis in metabolic disorders and reduce associated immune system dysregulation [8] or modulate the abundance of short-chain fatty acid-producing bacteria after a high adherence to enriched fruit and vegetable diets [6,51]. In humans, Ruminococcus degrade plant fibers and produce acetate and succinate as major end products [58]. *Ruminococcaceae* have been consistently described in the gut of healthy humans, with a significant reduction in abundance in patients with Crohn’s disease, suggesting a possible role in maintaining a healthy gut microbiome in these patients [59]. Moreover, in our intervention, the inferred microbiome metabolic data, derived from taxonomical analysis of the microbiota, indicated a significant reduction in rgpE-glucosyltransferase protein associated with the O-antigen lipopolysaccharide biosynthesis and a peptidoglycan recognition protein from the Toll and Imd signaling pathway (https://www.genome.jp/kegg/, accessed on 26 November 2021) in pigs fed the FV-supplemented diet relative to baseline levels, suggesting a diet induced modulation of the inflammatory response possibly by modulating the abundance of pro-inflammatory bacteria, as seen with the FV-supplemented group, where there was a significant reduction of potential pathogens associated with inflammatory disease (i.e., Campylobacteria and Gammaproteobacteria), suggesting an added benefit of FV diets that can be incorporated as a prophylactic approach for health maintenance. In addition, our FV-supplemented diet provided more fiber than the control diet for additional production of SCFA, which are linked to induced changes in immune activation. Therefore, taken together, our microbiome data indicated that a short-term two-week feeding with FV modulated the abundance and activity of specific butyrate-producing bacteria from *Erysipelotrichaceae*, *Ruminococcaceae* and *Bacteroidaceae* and fiber-degrading bacteria from *Lentisphaerae* that may contribute to the enrichment of more substrates for carbohydrate metabolism and the delivery of butyrate to the mucosa.

Inflammation is a normal response of the host defense that can become chronic if the provoking insult is not cleared [60]. Changes in the composition of the gut microbiome have been linked to the induction of chronic inflammation where there is migration of lymphocytes into inflamed non-lymphoid tissues that under normal conditions will not recruit these lymphocytes [61]. However, a reduction in inflammation [62] and diet-induced metabolic dysfunction [63] may result from the consumption of a healthy plant-based diet. A higher intake of vegetables has been linked to a lower WBC inflammatory profile through an altered gut microbiome, suggesting that a vegetable-rich diet is linked to a lower inflammation [64]. Our study provides broad molecular evidence that a combination of nine fruits and vegetables supplemented in the daily diet affects host genes related to the regulation of cellular movement, the inflammatory response, cell-to-cell signaling, and hematological system development that were predicted to be causally affected by the transcriptomic changes observed in WBC of pigs fed a supplemented FV diet. Specific functions associated with migration of cells, immune cell response, binding of mononuclear cells and lymphocytes, and adhesion were predicted to be downregulated (Z-score < 2.5). However, a stimulatory effect of the FV-supplemented diet on the development and differentiation of B cells was also detected. There was an upregulation of genes, associated with B-cell function, including two B cell restricted immunoglobulin heavy-chain constant regions, (*IGHA1*, *IGHG1*) and a B cell restricted immunoglobulin kappa-chain variable region (*IGKV7*). *CD79B*, the B-cell antigen receptor expressed by B cells [65], which is involved in antigen processing and presentation and also a B cell maturation marker [66], was upregulated in response to FV dietary intervention. Development of B-cell lineage was affected as *TNFRSF13C*, essential for B-cell development and function, was also positively regulated [67,68]. CD1A which is expressed by B cells in pigs and humans, but also by pig and human dendritic cells [69] and monocytes [70,71] is involved in lipid antigen presentation to T cells [72]. *LY86*, a positive regulator of signaling in response to LPS [73], is expressed in B cells and monocytes [74]. This gene expression pattern suggests that B cell number/function was positively affected by an FV-supplemented diet. In support of our observations, a polyphenol-rich variety of apples provided in the diet to healthy adults was also shown to induce changes in peripheral blood mononuclear cells (PBMC) gene expression with differential regulation of immunoglobulin-related genes that had roles in immunoglobulin production and B-cell mediated immunity [75]. Significant changes in the expression of genes associated with immune-related pathways were also detected in adults at risk of developing metabolic syndrome who received a freeze-dried blueberry-supplemented diet [76], thus demonstrating that controlled nutritional interventions have the potential to affect health through immune response modulation. However, more studies with larger sample numbers are needed to explain the molecular mechanism of action of different FV-enriched diets and its regulation of inflammation on metabolic diseases.

To further support the biological impact of the host transcriptome changes induced by our two-week dietary FV intervention, our DGE dataset showed a significant overlap with immunological datasets related to the induction of memory cell response and B-cell stimulation in previously defined functional profiles within the MSigDB database. Therefore, the data provide new molecular evidence that an FV-supplemented diet selectively suppresses the host inflammatory response and cellular movement through cytokine receptor signaling while stimulating B-cell development, suggesting that FV supplementation favorably affects the composition and function of the host intestinal microbiota with selective activation of humoral immune protection and reduced inflammation. However, the mechanism linking changes in the microbiome and WBC gene expression will need to be validated with focused functionality studies that evaluate the interaction of microbiome-derived metabolites or the direct effect of FV inherent bioactive components such as polyphenols.

## Data Availability

Supporting data provided in supplementary material.

## Supplementary Materials

The following are available online at https://www.mdpi.com/2072-6643/13/12/4350/s1, Figure S1: Body weight (Kgs) in pigs fed FV-supplemented or control diet, Figure S2: Bacteria taxa in baseline pigs. Figure S3: Inferred Metabolic Pathways associated with FV supplemented diets. Table S1: Dietary composition, Table S2: DGE in FV-supplemented group vs. Control, Table S3: DGE in FV-supplemented vs. baseline, Table S4: RT-PCR gene validation. Table S5: DGE not corrected by randomization in baseline samples. Table S6: Predicted biological functions affected with contributing genes and associated Z-scores. Table S7: Overlap with MSigDB datasets. Table S8: Non-chimeric reads for 16S rDNA analysis.
