# Correction: Solano-Aguilar et al. Fruit and Vegetable Supplemented Diet Modulates the Pig Transcriptome and Microbiome after a Two-Week Feeding Intervention. *Nutrients* 2021, *13*, 4350

**DOI:** 10.3390/nu14214513

**Published:** 2022-10-27

**Authors:** Gloria I. Solano-Aguilar, Sukla Lakshman, Jonathan Shao, Celine Chen, Ethiopia Beshah, Harry D. Dawson, Bryan Vinyard, Steven G. Schroeder, Saebyeol Jang, Aleksey Molokin, Joseph F. Urban

**Affiliations:** 1 U.S. Department of Agriculture, Northeast Area, Agricultural Research Service, Beltsville Human Nutrition, Research Center, Diet Genomics and Immunology Laboratory, Beltsville, MD 20705, USA; 2 Statistics and Bioinformatics Group, Agricultural Research Service, U.S. Department of Agriculture, Northeast Area, Beltsville, MD 20705, USA; 3 U.S. Department of Agriculture, Northeast Area, Agricultural Research Service, Beltsville Agricultural Research Center, Animal Genomics and Improvement Laboratory, Beltsville, MD 20705, USA

There was an error in the original publication [1].

In the original article, an incorrect IACUC protocol number was inadvertently included, and the article should have referenced protocol 19-016. All reported animal experiments and procedures were conducted in accordance with the guidelines established and approved by Beltsville Area Animal Care and Use Committee under protocol 19-016.

A correction has been made under Materials and Methods Section Under 2.1 Animals and Diets: all animal experiments and procedures were conducted in accordance with the guidelines established and approved by Beltsville Area Animal Care and Use Committee under protocol 19-016.

A correction has been made under Institutional Review Board Statement: all animal experiments and procedures were conducted in accordance with the guidelines established and approved by Beltsville Area Animal Care and Use Committee under protocol 19-016.

The authors apologize for any inconvenience caused and state that the scientific conclusions are unaffected. This correction was approved by the Academic Editor. The original publication has also been updated.
