# Supplementary material for: Fruit and Vegetable Supplemented Diet Modulates the Pig Transcriptome and Microbiome after a Two-Week Feeding Intervention
Source: Nutrients. 2021 Dec 2;13(12):4350. doi: 10.3390/nu13124350 (PMC8703502; doi:10.3390/nu13124350)
Supplement: Supplementary file 1 [file nutrients-13-04350-s001.zip › supplementary/nutrients-1408016-supplementary-figures.pdf]

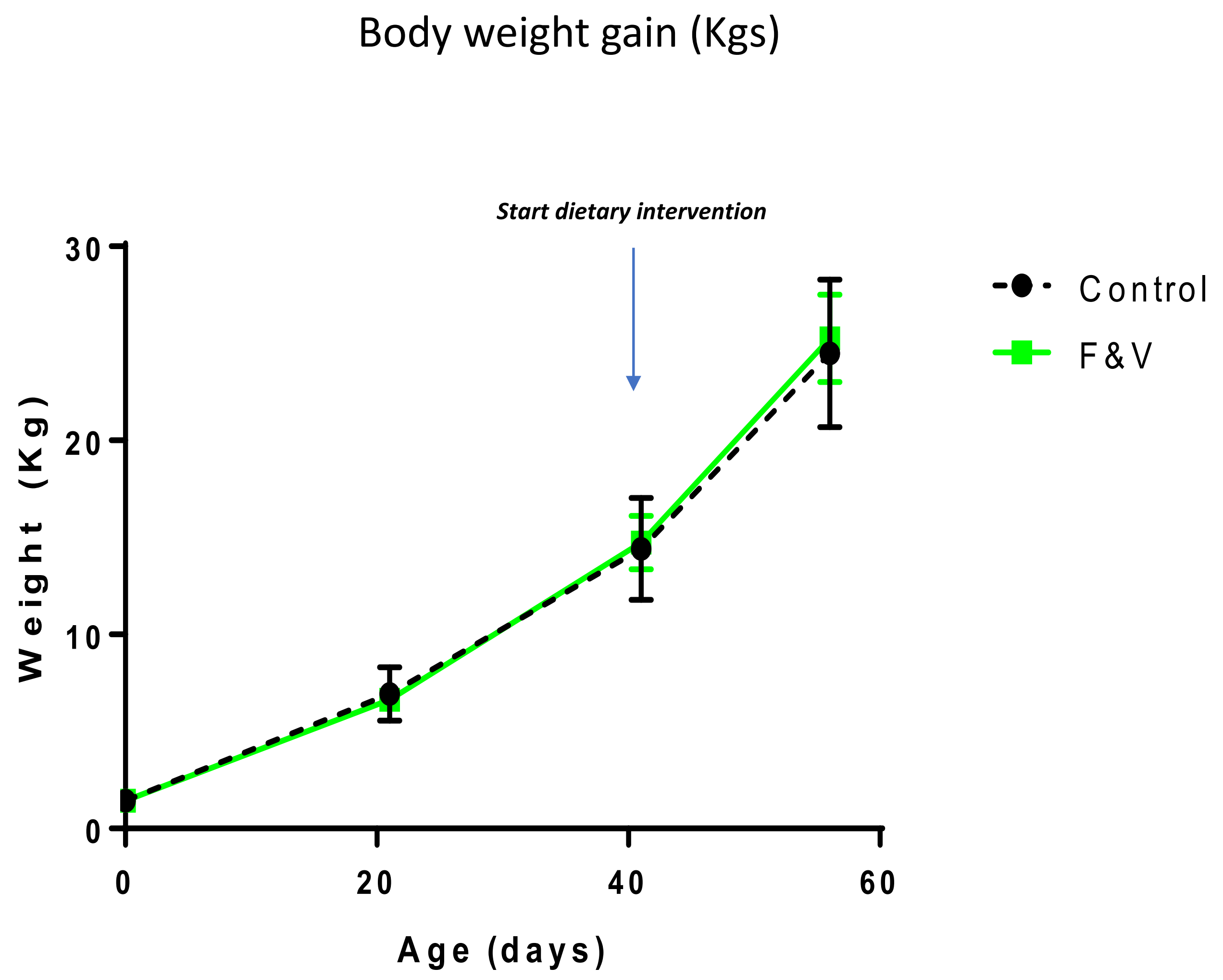
Body weight gain (Kgs)
Start dietary intervention
Weight (Kg)
Age (days)
0
10
20
30
0
20
40
60
Control
F&V

Figure S1

Lefse representing differentially abundant taxa among baseline samples

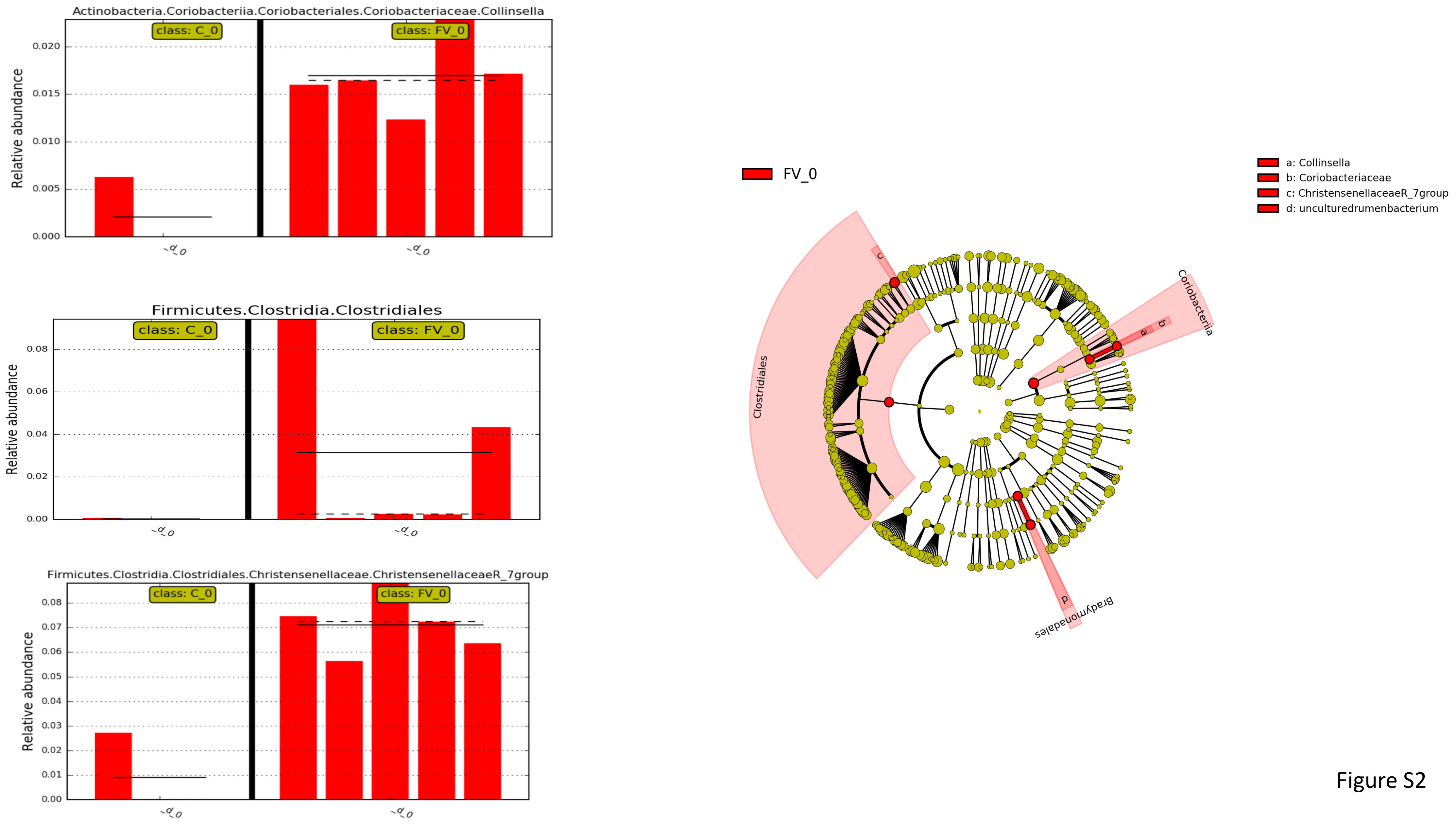

Figure S2

PICRUSt2-predicted KEGG metabolic pathways after 2 Wks FV-supplemented diet

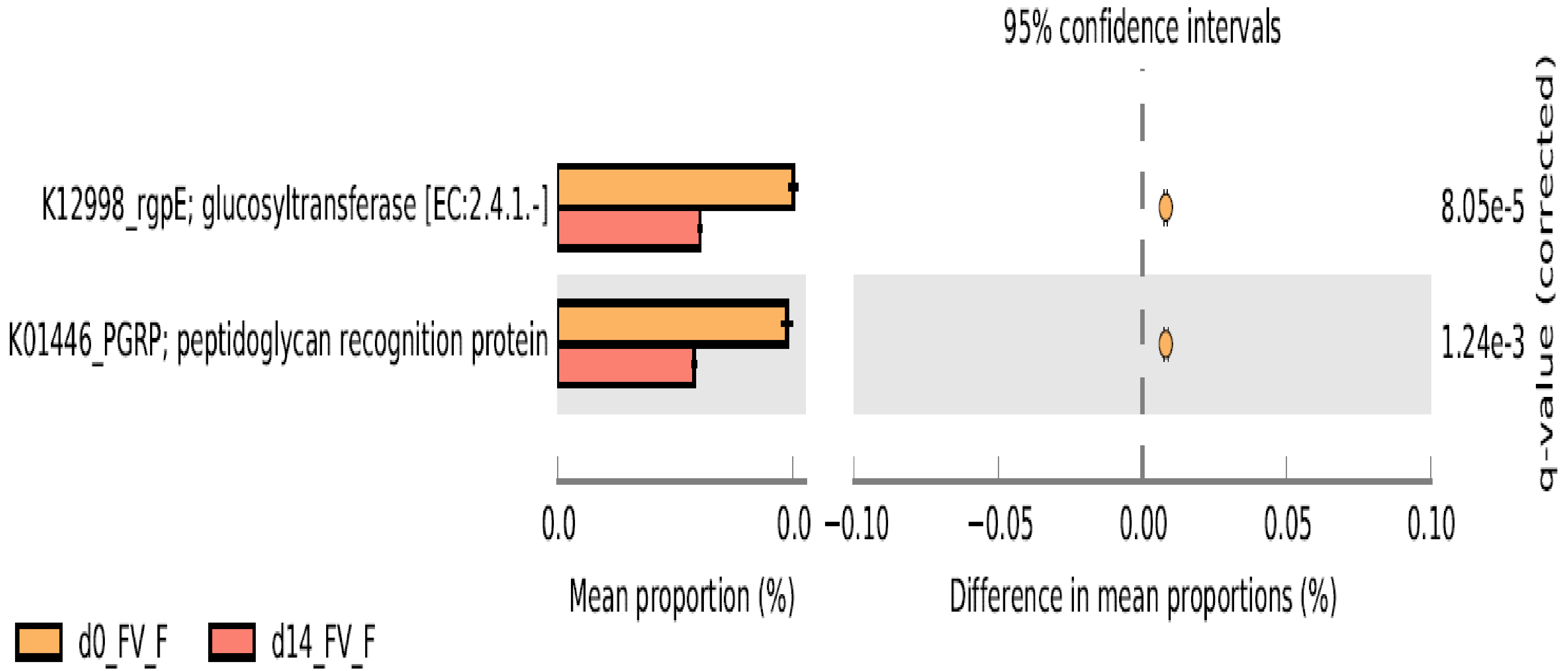

Figure S3
