# Supplementary material for: Fruit and Vegetable Supplemented Diet Modulates the Pig Transcriptome and Microbiome after a Two-Week Feeding Intervention
Source: Nutrients. 2021 Dec 2;13(12):4350. doi: 10.3390/nu13124350 (PMC8703502; doi:10.3390/nu13124350)
Supplement: Supplementary file 1 [file nutrients-13-04350-s001.zip › supplementary/nutrients-1408016-supplementary.pdf]

Table S1. Analysis of fruit and vegetable mix and chicken nutrient composition

Nutrient	Per 100 g lyophilized fruits	Per 100 g lyophilized veggies	Per 100 g lyophilized chicken
Protein, g/ 100 g	4.42	27.04	69.8
Carbohydrate, g/ 100 g	69.54	43.77	<0.5
Fat, g/ 100 g	5.68	6.42	25.44
Ash, g/ 100 g	2.05	13.97	3.86
Moisture, g/ 100 g	18.31	8.8	2.03
Total dietary Fiber, g/ 100 g	18.3	29.6	-

Lyophilized fruit and vegetable mix was analyzed by Eurofins Scientific Inc.Des Moines, IA

Pig Diets

Nutrient g /Kg diet	Control	FV
Protein	160	160
Carbohydrate	484.6	504.1
Fat	60	60
Fiber	26.7	29.9
Total Kcal/Kg diet	3118.4	3196.4
**Ingredients (g)**		
Corn	715.58	695.22
46% Soybean Meal	221.35	192.13
Chicken_liophilized	0	13.17
Fruit Mix	0	25.12
Vegetable Mix	0	15.18
Salt	4	4
Calcium Carbonate	10.21	10.28
Swine Vitamins_Minerals	3	3
Methionine	0.781	0.922
Lysine	2.629	3.095
Threonine	0.585	0.799

Soybean oil	30.69	26.14
Monocalcium phosphate	11.16	10.92
	999.98	999.97

Table S2-Differential gene expression in whole blood of pigs supplemented FV vs Control diet after 2 weeks

Gene ID NR	Gene ID Ensembl	Subset NR FV vs C	Subset Ensembl FV vs C	FC[a]	padj
IGKV7	ENSSSCG00000033808	0	1	5.4	2.84E-04
IGHG1**	IGHG1.01	1	0	5.2	4.94E-02
ZBTB32	ZBTB32	0	1	4.3	3.60E-04
F12	F12	1	0	3.9	3.55E-02
LY86	LY86	1	1	3.7	7.38E-04
IGLL5L*	IGLL5L	1	0	3.6	9.63E-03
FCRL5	FCRL5	1	0	3.6	1.65E-05
SPIB	ENSSSCG00000034211	0	1	3.4	8.03E-04
CD1A	CD1A	1	0	3.3	1.07E-04
MZB1	MZB1	1	0	3.2	8.50E-02
RGS19	RGS19	1	0	3.1	9.61E-02
CD79B	CD79B	0	1	3.1	1.50E-02
TNFRSF13C	TNFRSF13C	1	1	3.1	3.70E-03
IGHA1	IGHA1	1	0	3.0	3.41E-02
PRR5	PRR5	0	1	2.8	4.39E-02
ND3	ND3	0	1	2.8	8.05E-02
ID3	ID3	1	1	2.8	1.78E-02
POU2AF1	POU2AF1	1	1	2.7	2.93E-02
CD72	CD72	1	0	2.6	8.10E-02
ATP8	ATP8	0	1	2.6	7.41E-02
ND1	ND1	1	1	2.6	9.61E-02
IGJ	IGJ	1	0	2.5	3.31E-02
SLA-DQA1	SLA-DQA1	1	0	2.5	5.42E-03
JCHAIN	JCHAIN	0	1	2.4	3.62E-02
CMTM7	ENSSSCG00000011234	0	1	2.4	1.50E-02
CD79A	CD79A	1	0	2.4	2.84E-02
TRAF2	TRAF2	1	0	2.4	7.39E-02
SLA-DRA	SLA-DRA	1	0	2.4	1.00E-02
SH3TC1	ENSSSCG00000008722	0	1	2.4	2.88E-02
SLA-DOB	SLA-DOB	1	0	2.4	9.61E-02
SLA-DQA1	ENSSSCG00000001456	0	1	2.3	1.46E-02
PRDX4	PRDX4	1	1	2.3	6.28E-02

SLA-DRA	HLA-DRA	0	1	2.3	1.53E-02
SLA-DRB2	SLA-DRB2	1	0	2.3	9.68E-02
CD74	CD74	1	0	2.3	3.47E-02
CRIP1	CRIP1	1	0	2.3	8.50E-02
MCRIP1	MCRIP1	0	1	2.2	8.28E-02
MS4A1	MS4A1	0	1	2.2	2.26E-02
VAMP8	VAMP8	0	1	2.1	7.04E-02
THY1	THY1	1	1	2.0	8.00E-02
EMP3	EMP3	1	0	2.0	9.61E-02
TRDC	TRDC	0	1	2.0	9.40E-02
SLA-DQB1	SLA-DQB1	0	1	2.0	9.94E-02
SLA-DRB1	ENSSSCG00000001455	0	1	1.9	9.56E-02
PIK3IP1	PIK3IP1	0	1	-1.9	7.41E-02
ITGAX	ITGAX	0	1	-1.9	7.41E-02
KLF13	KLF13	1	0	-2.0	6.64E-02
APOBR	ENSSSCG00000033623	0	1	-2.1	7.41E-02
STEAP4	STEAP4	0	1	-2.1	9.23E-02
SIGLEC14	SIGLEC14	1	0	-2.1	8.50E-02
ITGAM	ITGAM	1	0	-2.2	8.10E-02
IL13RA1	IL13RA1	0	1	-2.2	9.41E-02
TXNIP	TXNIP	1	0	-2.3	6.64E-02
UBE2H	UBE2H	0	1	-2.3	7.41E-02
CXCR4	CXCR4	0	1	-2.3	2.28E-02
C5AR1	C5AR1	0	1	-2.3	9.41E-02
TINAGL1	TINAGL1	0	1	-2.3	7.28E-02
FAM49A	FAM49A	0	1	-2.3	7.41E-02
SGK1	SGK1	0	1	-2.4	9.00E-02
ZCCHC6	ZCCHC6	0	1	-2.4	7.41E-02
ERAP1	ERAP1	0	1	-2.4	3.43E-02
MLXIP	MLXIP	1	1	-2.4	4.94E-02
NOTCH2	NOTCH2	1	1	-2.6	7.39E-02
LILR1B1*	ENSSSCG00000028802	0	1	-2.7	8.91E-02
PELI1	PELI1	0	1	-2.7	5.94E-02
EP300	EP300	1	1	-2.7	9.61E-02

GPCPD1	GPCPD1	0	1	-2.7	7.04E-02
ACSL1	ACSL1	0	1	-2.7	6.16E-02
SORL1	SORL1	1	1	-2.7	2.75E-02
DUSP1	DUSP1	1	1	-2.8	2.43E-02
ACSL4	ACSL4	0	1	-2.8	3.43E-02
SCML4	SCML4	0	1	-2.8	3.21E-02
FOXO3	FOXO3	1	1	-2.9	4.14E-02
IL7R	IL7R	0	1	-2.9	1.46E-02
UBE2O	UBE2O	0	1	-2.9	9.94E-02
NIN	NIN	0	1	-3.0	8.18E-02
THBS1	THBS1	1	1	-3.0	8.50E-02
VNN1	VNN1	0	1	-3.1	7.41E-02
ADAM19	ADAM19	1	1	-3.2	5.16E-03
LYST	LYST	0	1	-3.2	7.20E-02
WDFY3	WDFY3	0	1	-3.2	5.28E-02
IL6ST	IL6ST	0	1	-3.3	9.54E-02
ATP8B4	ATP8B4	1	0	-3.4	6.64E-02
CRISPLD2	CRISPLD2	1	1	-3.8	3.47E-02
CEBPB	CEBPB	1	1	-3.9	1.71E-03
FRY	FRY	1	0	-4.0	2.43E-02
MXD1	MXD1	0	1	-4.2	1.10E-03
NCOA4	NCOA4	0	1	-4.3	1.46E-02
DGAT2	DGAT2	0	1	-4.5	1.33E-03
MEGF9	MEGF9	0	1	-4.5	7.41E-02
TGM3	TGM3	1	1	-5.0	2.79E-04
IL1R2	IL1R2	0	1	-5.3	8.03E-04
FKBP5	FKBP5	0	1	-5.4	2.11E-06
SSH2	SSH2	0	1	-5.6	3.43E-02

* = pig specific gene

** = gene name changed from IGHG1.01 to IGHG1

[a] FC from NR datbase is used for reporting unless not available replaced by Ensembl database

Table S3-Differential gene expression in whole blood of pigs fed FV-supplemented diets after two weeks (compared gainst baseline)

Gene ID NR	Gene ID Ensembl	Subset ofNR	Subset of Ensembl	FC [a]	padj
RGS19	RGS19	1	0	3.5	7.00E-03
IGKV7	ENSSSCG00000033808	0	1	3.3	1.16E-03
IGHG1	IGHG1.01	1	0	3.0	7.24E-02
ZBTB32	ZBTB32	0	1	2.9	1.23E-03
GP1BB	GP1BB	1	0	2.8	9.18E-02
LY86	LY86	1	1	2.7	1.08E-03
IGLL5L1*	IGLL5L	1	0	2.6	1.20E-02
SPIB	ENSSSCG00000034211	0	1	2.5	1.16E-03
FCRL5	FCRL5	1	0	2.5	8.79E-05
TNFRSF13C	TNFRSF13C	1	1	2.5	1.70E-03
CD79B	CD79B	0	1	2.5	1.15E-02
MZT2B	ENSSSCG00000010104	0	1	2.5	3.20E-02
CD1A	CD1A	1	0	2.4	1.57E-04
POU2AF1	POU2AF1	1	1	2.4	5.39E-03
FCRL5	ENSSSCG00000026210	0	1	2.4	2.60E-04
MZB1	MZB1	0	1	2.4	5.09E-02
ND3	ND3	0	1	2.4	2.48E-02
IGHA1	IGHA1	1	0	2.4	1.71E-02
F12	F12	0	1	2.4	6.97E-02
BCL7C	BCL7C	1	1	2.4	5.96E-02
ND1	ND1	1	1	2.4	2.29E-02
FAM173A	FAM173A	0	1	2.3	5.77E-02
IGH@	IGH@	1	0	2.3	6.05E-02
GPR146	GPR146	1	0	2.3	7.82E-02
EIF4EBP1	EIF4EBP1	0	1	2.3	3.01E-02
SIGLEC5L1	SIGLEC5L1	1	0	2.3	9.93E-02
EMC6	EMC6	0	1	2.3	4.87E-02
IGKC	ENSSSCG00000036224	0	1	2.2	5.65E-02
NDUFS8	NDUFS8	0	1	2.2	3.01E-02
CD1A	ENSSSCG00000025644	0	1	2.2	1.16E-03
ITGAD	ITGAD	1	0	2.2	5.21E-02
GADD45GIP1	GADD45GIP1	1	0	2.2	6.63E-02

ID3	ID3	1	1	2.2	1.71E-02
PRR5	PRR5	0	1	2.1	4.23E-02
MVD	MVD	1	0	2.1	9.18E-02
FHL3	FHL3	1	0	2.1	8.95E-02
JPT1	JPT1	0	1	2.1	4.98E-02
COA1	ENSSSCG00000027491	0	1	2.1	9.45E-02
GATD1	GATD1	0	1	2.1	9.11E-02
ANGPTL4	ANGPTL4	0	1	2.1	4.97E-02
TMEM147	TMEM147	0	1	2.1	4.84E-02
ADI1	ADI1	0	1	2.1	6.11E-02
FAAP20	FAAP20	1	0	2.1	3.80E-02
THAP3	THAP3	0	1	2.0	9.83E-02
SLA-DRB2	SLA-DRB2	1	0	2.0	3.55E-02
PSMG4	ENSSSCG00000036988	0	1	2.0	5.34E-02
GTF3A	GTF3A	0	1	2.0	7.90E-02
PLA2G16	PLA2G16	1	0	2.0	6.99E-02
SLA-DOA	SLA-DOA	1	1	2.0	6.58E-02
DNASE1L1	DNASE1L1	1	1	2.0	7.82E-02
CD72	CD72	0	1	2.0	5.27E-02
IGJ	IGJ	1	0	2.0	3.13E-02
NFKBIB	NFKBIB	0	1	2.0	4.84E-02
NOP16	NOP16	1	0	2.0	8.73E-02
SSNA1	SSNA1	1	0	2.0	9.45E-02
POU2F2	POU2F2	0	1	2.0	6.07E-02
SLA-DQA1	SLA-DQA1	1	0	2.0	8.45E-03
UBE2S	UBE2S	1	0	2.0	9.60E-02
PRDX5	PRDX5	0	1	2.0	6.83E-02
SH3TC1	ENSSSCG00000008722	0	1	2.0	1.96E-02
LGALS1	LGALS1	1	0	2.0	9.62E-02
PRDX4	PRDX4	1	1	1.9	3.80E-02
TRAF2	TRAF2	1	0	1.9	6.02E-02
MECP2	MECP2	0	1	1.9	8.18E-02
SLA-DRA	SLA-DRA	1	0	1.9	1.25E-02
RNASEH2C	RNASEH2C	0	1	1.9	9.20E-02

ARFGAP1	ARFGAP1	0	1	1.9	6.97E-02
JCHAIN	JCHAIN	0	1	1.9	3.53E-02
CRIP1	CRIP1	1	0	1.9	4.75E-02
VAMP8	VAMP8	0	1	1.9	1.96E-02
ORMDL2	ENSSSCG00000000365	0	1	1.9	9.61E-02
SELENOH	SELENOH	1	0	1.9	9.81E-02
CMTM7	ENSSSCG00000011234	0	1	1.9	2.00E-02
CD79A	CD79A	0	1	1.9	3.84E-02
MRPL52	MRPL52	0	1	1.9	7.44E-02
ND2	ND2	0	1	1.9	7.41E-02
ST6GALNAC4	ST6GALNAC4	0	1	1.9	2.62E-02
MRPL12	MRPL12	1	0	1.9	8.93E-02
NOP53	NOP53	1	1	1.9	6.58E-02
MCRIP1	MCRIP1	0	1	1.9	4.43E-02
ATP6	ATP6	0	1	1.9	7.90E-02
DUSP22	DUSP22	1	1	1.9	4.75E-02
ZGPAT	ZGPAT	0	1	1.9	3.53E-02
FGD2	FGD2	0	1	1.9	7.81E-02
SLC35C2	SLC35C2	0	1	1.9	7.44E-02
ND6	ND6	0	1	1.9	8.01E-02
RPL8	RPL8	0	1	1.9	3.53E-02
BLK	BLK	0	1	1.9	4.84E-02
C21ORF33	C21ORF33	1	0	1.8	8.50E-02
THY1	THY1	1	1	1.8	2.91E-02
MIF	MIF	1	1	1.8	6.63E-02
ND4	ND4	0	1	1.8	7.44E-02
TUBB2B	TUBB2B	0	1	1.8	3.01E-02
CKS1B	CKS1B	0	1	1.8	7.44E-02
PGLS	PGLS	0	1	1.8	4.84E-02
TSPAN31	TSPAN31	1	0	1.8	4.75E-02
HSPB1	HSPB1	1	0	1.8	8.33E-02
CAPN10	CAPN10	1	0	1.8	7.82E-02
GATSL3	GATSL3	1	0	1.8	7.25E-02
RPS5	RPS5	0	1	1.8	3.30E-02

POFUT2	POFUT2	0	1	1.8	7.44E-02
CYTB	CYTB	0	1	1.8	7.33E-02
SLA-DQB1	SLA-DQB1	0	1	1.8	3.31E-02
RNH1	RNH1	1	1	1.8	6.58E-02
ENG	ENG	0	1	1.8	9.89E-02
BAK1	BAK1	1	0	1.8	6.63E-02
TUBB3	TUBB3	1	0	1.8	9.62E-02
PSENEN	ENSSSCG00000032059	0	1	1.8	9.94E-02
GMFG	GMFG	0	1	1.8	9.15E-02
CYB561A3	CYB561A3	0	1	1.8	5.48E-02
ZC3H3	ZC3H3	1	0	1.8	9.81E-02
MRPS34	MRPS34	0	1	1.8	6.05E-02
JUND	JUND	1	0	1.8	5.21E-02
GSTP1	GSTP1	1	1	1.8	5.34E-02
LTB	LTB	1	0	1.8	9.34E-02
XBP1	XBP1	0	1	1.7	5.85E-02
SLA-DRB1	SLA-DRB1	1	0	1.7	6.58E-02
EPN1	EPN1	1	0	1.7	6.58E-02
CASTOR1	CASTOR1	0	1	1.7	7.90E-02
TP53I11	TP53I11	0	1	1.7	8.07E-02
ATP8	ATP8	0	1	1.7	8.64E-02
PPDPF	PPDPF	1	0	1.7	9.55E-02
MS4A1	MS4A1	0	1	1.7	3.83E-02
ZC3H12D	ZC3H12D	1	0	1.7	9.18E-02
TRAPPC1	TRAPPC1	1	0	1.7	7.82E-02
SELENOW	SELENOW	1	0	1.7	9.81E-02
FUNDC2	FUNDC2	0	1	1.7	6.05E-02
SIGIRR	SIGIRR	0	1	1.7	6.63E-02
COX3	COX3	0	1	1.7	9.15E-02
CD74	CD74	0	1	1.7	5.04E-02
RPL28	RPL28	1	0	1.7	6.76E-02
IGHD	IGHD	1	0	1.7	7.74E-02
EEF1D	EEF1D	0	1	1.7	6.64E-02
EIF3F	EIF3F	1	0	1.7	7.88E-02

UBE2L6	UBE2L6	0	1	1.7	9.12E-02
CYC1	CYC1	0	1	1.7	5.10E-02
NDUFB9	NDUFB9	1	0	1.7	8.74E-02
VAV2	VAV2	0	1	1.7	6.76E-02
ATP5C1	ATP5C1	1	0	1.7	6.63E-02
EIF5A	EIF5A	0	1	1.7	5.10E-02
EMP3	EMP3	0	1	1.7	6.07E-02
ILK	ILK	0	1	1.7	5.04E-02
S100A4	S100A4	0	1	1.7	9.61E-02
RPS14	ENSSSCG00000031370	0	1	1.7	8.01E-02
SLA-DMB	SLA-DMB	1	0	1.6	9.64E-02
RPUSD1	RPUSD1	0	1	1.6	8.01E-02
EIF3G	EIF3G	1	0	1.6	9.94E-02
POLR2E	POLR2E	0	1	1.6	9.89E-02
RPS26	ENSSSCG00000033697	0	1	1.6	9.89E-02
CDK2AP2	CDK2AP2	0	1	1.6	9.20E-02
LY6E	LY6E	1	0	1.6	7.64E-02
TRIM34	TRIM34	1	0	1.6	8.05E-02
RPS14	RPS14	1	0	1.6	9.81E-02
RPL18	RPL18	0	1	1.6	8.95E-02
EEF1G	ENSSSCG00000013064	0	1	1.6	9.61E-02
RPL7A	RPL7A	1	1	1.6	6.97E-02
RPL10	RPL10	0	1	1.6	9.20E-02
TRDC	TRDC	0	1	1.6	9.20E-02
ARPC1B	ARPC1B	0	1	1.6	9.43E-02
BTK	BTK	0	1	1.6	7.44E-02
FAM89B	FAM89B	1	0	1.6	7.34E-02
TAGLN2	TAGLN2	1	0	1.6	9.18E-02
ABHD17A	ABHD17A	0	1	1.6	6.76E-02
SEPT15	SEPT15	1	0	1.6	9.55E-02
RPL13A	RPL13A	1	0	1.6	7.82E-02
PLAC8	PLAC8	1	0	1.6	9.60E-02
ATP6V0B	ATP6V0B	1	1	1.6	9.18E-02
PSMA7	PSMA7	0	1	1.6	9.89E-02

PSME1	PSME1	1	0	1.5	8.95E-02
RPS20	RPS20	0	1	1.5	9.21E-02
S100A10	S100A10	0	1	1.5	9.73E-02
RPS9	RPS9	0	1	1.5	7.44E-02
MLF2	MLF2	0	1	1.5	9.78E-02
MXD4	MXD4	1	0	1.5	9.99E-02
FLT3LG	ENSSSCG00000034448	0	1	1.5	9.91E-02
RPL35	RPL35	0	1	1.5	7.23E-02
RPL32	RPL32	1	0	1.5	9.77E-02
KLF2	KLF2	1	0	1.5	8.84E-02
PITPNM1	PITPNM1	0	1	1.5	9.20E-02
CTBP1	CTBP1	1	0	1.5	9.93E-02
NAP1L1	ENSSSCG00000000522	0	1	1.5	7.44E-02
RPL39	ENSSSCG00000030849	0	1	1.4	9.85E-02
JAK1	JAK1	1	1	-1.4	8.93E-02
MSN	MSN	0	1	-1.5	9.89E-02
PREX1	PREX1	0	1	-1.5	8.95E-02
PIK3IP1	PIK3IP1	0	1	-1.5	8.44E-02
CYBB	CYBB	1	0	-1.5	9.81E-02
IFNAR1	IFNAR1	1	1	-1.6	7.45E-02
ITGAL	ITGAL	0	1	-1.6	6.97E-02
CD46	ENSSSCG00000038506	0	1	-1.6	6.97E-02
TOP1	TOP1	0	1	-1.6	9.20E-02
IVNS1ABP	IVNS1ABP	0	1	-1.6	8.54E-02
ACLY	ACLY	0	1	-1.6	4.97E-02
IQGAP1	IQGAP1	1	0	-1.6	8.93E-02
TRIM4	TRIM4	0	1	-1.7	8.01E-02
CCPG1	CCPG1	0	1	-1.7	7.94E-02
FAM105A	FAM105A	0	1	-1.7	8.44E-02
LDLR	LDLR	0	1	-1.7	9.15E-02
PTPRC	PTPRC	0	1	-1.7	9.11E-02
RMND5A	RMND5A	0	1	-1.7	8.10E-02
LGALS8	LGALS8	0	1	-1.7	8.44E-02
PICALM	PICALM	1	1	-1.7	5.44E-02

WIPF1	WIPF1	0	1	-1.7	6.50E-02
PPP6R3	PPP6R3	0	1	-1.7	9.89E-02
KLF13	KLF13	1	0	-1.7	4.75E-02
ERAP2	ERAP2	0	1	-1.7	7.81E-02
KMT2D	KMT2D	1	0	-1.7	5.44E-02
IFIT2	IFIT2	1	1	-1.7	7.94E-02
PRKCH	PRKCH	0	1	-1.7	9.15E-02
CXCR4	CXCR4	0	1	-1.7	4.84E-02
KIAA1191	ENSSSCG00000014060	0	1	-1.7	9.20E-02
MYH9	MYH9	1	1	-1.7	6.10E-02
SEMA7A	SEMA7A	0	1	-1.7	8.01E-02
AKAP13	AKAP13	1	0	-1.7	1.92E-02
ITGB1	ITGB1	0	1	-1.8	9.53E-02
USP9X	USP9X	0	1	-1.8	6.76E-02
CYBRD1	CYBRD1	0	1	-1.8	8.22E-02
IL13RA1	IL13RA1	1	1	-1.8	7.82E-02
CEP250	CEP250	0	1	-1.8	9.43E-02
C5AR1	C5AR1	0	1	-1.8	9.20E-02
UBE2B	UBE2B	0	1	-1.8	7.44E-02
HERC3	HERC3	0	1	-1.8	3.38E-02
UBE2H	UBE2H	0	1	-1.8	6.76E-02
CSF2RB	CSF2RB	1	1	-1.8	7.82E-02
IFIT1	IFIT1	0	1	-1.8	7.33E-02
DOCK8	DOCK8	0	1	-1.8	9.78E-02
STEAP4	STEAP4	0	1	-1.8	4.66E-02
TGFBI	TGFBI	0	1	-1.8	7.44E-02
DNMT1	DNMT1	1	1	-1.8	6.10E-02
MLXIP	MLXIP	1	1	-1.9	6.42E-02
TXNIP	TXNIP	1	0	-1.9	5.23E-02
NEAT1	NEAT1	1	0	-1.9	7.13E-02
PRKD3	PRKD3	0	1	-1.9	9.48E-02
FAM49A	FAM49A	0	1	-1.9	4.93E-02
BRWD1	BRWD1	1	0	-1.9	7.24E-02
ZCCHC6	ZCCHC6	0	1	-1.9	5.93E-02

MMD	MMD	0	1	-1.9	7.95E-02
HIST1H1D	HIST1H1D	0	1	-1.9	9.20E-02
DGAT2	DGAT2	1	0	-1.9	9.57E-02
CPD	CPD	0	1	-1.9	9.02E-02
CLK1	CLK1	0	1	-1.9	9.93E-02
GPCPD1	GPCPD1	0	1	-1.9	7.44E-02
NCOA6	NCOA6	0	1	-2.0	8.01E-02
PAG1	PAG1	1	1	-2.0	6.63E-02
TRIB1	TRIB1	0	1	-2.0	9.93E-02
ADNP	ADNP	0	1	-2.0	9.91E-02
SETD5	SETD5	1	1	-2.0	6.06E-02
ARRDC4	ARRDC4	1	1	-2.0	9.18E-02
SVIL	SVIL	0	1	-2.0	3.01E-02
ADCY7	ADCY7	0	1	-2.0	6.90E-02
AHCTF1	AHCTF1	0	1	-2.0	7.33E-02
ZNF217	ZNF217	0	1	-2.0	9.11E-02
ZC3H13	ZC3H13	0	1	-2.0	5.45E-02
BCL2L1	BCL2L1	0	1	-2.0	7.82E-02
HIATL1	HIATL1	1	0	-2.0	8.93E-02
UBR2	UBR2	0	1	-2.0	4.87E-02
TRIB2	TRIB2	0	1	-2.0	4.84E-02
ELMSAN1	ELMSAN1	1	0	-2.0	6.58E-02
TCF20	TCF20	0	1	-2.0	4.49E-02
CEBPB	CEBPB	1	1	-2.0	6.10E-02
USP34	USP34	0	1	-2.0	8.18E-02
SERINC5	SERINC5	0	1	-2.0	6.76E-02
GP91-PHOX	GP91-PHOX	0	1	-2.0	9.83E-02
EIF4G3	EIF4G3	0	1	-2.0	2.95E-02
MDN1	MDN1	1	1	-2.0	7.82E-02
UBE2O	UBE2O	1	1	-2.0	8.88E-02
CHST15	CHST15	0	1	-2.1	4.23E-02
NFATC3	NFATC3	0	1	-2.1	8.95E-02
RIF1	RIF1	1	0	-2.1	9.81E-02
ACSL1	ACSL1	0	1	-2.1	4.84E-02

WDFY3	WDFY3	0	1	-2.1	9.20E-02
CPEB4	CPEB4	1	1	-2.1	6.58E-02
VCPIP1	VCPIP1	0	1	-2.1	9.15E-02
MED13	MED13	1	0	-2.1	9.60E-02
B4GALT5	B4GALT5	0	1	-2.1	6.76E-02
BIRC6	BIRC6	1	1	-2.1	3.89E-02
MFSD6	MFSD6	0	1	-2.1	6.80E-02
ZMIZ1	ZMIZ1	1	0	-2.1	8.33E-02
CCNL1	CCNL1	0	1	-2.1	4.93E-02
VNN1	VNN1	0	1	-2.1	9.21E-02
KMT2E	KMT2E	0	1	-2.1	4.84E-02
TNIK	TNIK	0	1	-2.1	9.15E-02
MFSD14B	MFSD14B	0	1	-2.1	7.49E-02
ADAM19	ADAM19	1	1	-2.1	3.13E-02
ERAP1	ERAP1	0	1	-2.1	1.26E-02
NLRP3	NLRP3	0	1	-2.1	4.66E-02
SGK1	SGK1	0	1	-2.1	2.48E-02
IQGAP2	IQGAP2	1	1	-2.1	5.44E-02
PHF2	PHF2	1	1	-2.1	6.58E-02
ARID1B	ARID1B	0	1	-2.2	6.80E-02
SORL1	SORL1	1	1	-2.2	1.92E-02
TGM3	TGM3	1	1	-2.2	5.44E-02
TACC1	ENSSSCG00000015814	0	1	-2.2	5.10E-02
PPM1H	PPM1H	1	0	-2.2	6.93E-02
TNFAIP3	TNFAIP3	1	0	-2.2	9.81E-02
SATB1	SATB1	0	1	-2.2	9.89E-02
ATP8B4	ATP8B4	1	0	-2.3	8.74E-02
PER1	PER1	1	1	-2.3	7.82E-02
HIPK1	HIPK1	0	1	-2.3	3.56E-02
TMEM43	TMEM43	0	1	-2.3	3.72E-02
FOXO3	FOXO3	1	1	-2.3	2.81E-02
JMJD1C	JMJD1C	0	1	-2.3	5.20E-02
USP32	USP32	1	0	-2.3	5.53E-02
CRISPLD2	CRISPLD2	1	1	-2.3	7.88E-02

PRRC2C	PRRC2C	1	1	-2.3	5.53E-02
SLFN14	SLFN14	0	1	-2.3	9.20E-02
PLXNC1	PLXNC1	0	1	-2.3	7.78E-02
REV3L	REV3L	1	0	-2.3	8.95E-02
BCL6	BCL6	1	1	-2.3	7.13E-02
SP3	SP3	0	1	-2.3	5.48E-02
ACSL4	ACSL4	0	1	-2.4	1.26E-02
THBS1	THBS1	1	1	-2.4	5.63E-02
SECISBP2L	SECISBP2L	1	0	-2.4	8.93E-02
SGMS1	SGMS1	0	1	-2.4	5.97E-02
IL7R	IL7R	0	1	-2.4	5.47E-03
GIGYF2	GIGYF2	0	1	-2.4	2.00E-02
NOTCH2	NOTCH2	1	1	-2.4	1.27E-02
NIN	NIN	0	1	-2.4	3.53E-02
ITGA4	ITGA4	1	1	-2.4	6.63E-02
SPIN1	SPIN1	0	1	-2.5	3.53E-02
MARCH3	MARCH3	1	0	-2.5	7.82E-02
CD101	CD101	1	0	-2.5	9.64E-02
PELI1	PELI1	0	1	-2.5	5.48E-03
BPTF	ENSSSCG00000033528	0	1	-2.5	3.01E-02
NCOA2	NCOA2	1	0	-2.5	5.21E-02
LTBP1	LTBP1	1	0	-2.5	9.64E-02
ADNP2	ADNP2	1	0	-2.5	8.93E-02
KIAA1109	KIAA1109	0	1	-2.5	8.01E-02
KCNJ2	KCNJ2	0	1	-2.5	9.15E-02
SCML4	SCML4	0	1	-2.6	5.47E-03
EIF2AK4	EIF2AK4	1	0	-2.6	7.82E-02
EP300	EP300	1	1	-2.6	1.27E-02
LRRK2	LRRK2	0	1	-2.6	4.23E-02
ASXL2	ASXL2	1	0	-2.6	8.66E-02
RASA2	RASA2	0	1	-2.6	8.21E-02
MXD1	MXD1	0	1	-2.6	1.06E-02
ZNF236	ZNF236	1	0	-2.7	9.45E-02
FAM46A	FAM46A	0	1	-2.7	3.01E-02

GVIN1	GVIN1	1	0	-2.7	7.82E-02
HIVEP2	HIVEP2	1	0	-2.7	5.44E-02
FKBP5	FKBP5	0	1	-2.8	9.35E-04
LNPEP	LNPEP	0	1	-2.8	2.77E-02
MED13L	MED13L	1	0	-2.8	7.82E-02
EPB41	EPB41	0	1	-2.8	3.01E-02
AFF4	AFF4	1	0	-2.8	6.10E-02
SYNE1	SYNE1	1	0	-2.9	6.02E-02
IL1R2	IL1R2	0	1	-2.9	1.21E-02
VCAN	VCAN	0	1	-2.9	1.26E-02
NCOA4	NCOA4	0	1	-2.9	1.95E-02
LYST	LYST	0	1	-2.9	1.26E-02
FRY	FRY	1	0	-2.9	1.71E-02
FN1	FN1	1	0	-3.0	7.93E-02
MEGF9	MEGF9	0	1	-3.0	5.48E-02
PUM1	PUM1	0	1	-3.2	2.70E-02
KMT2C	KMT2C	1	1	-3.2	6.06E-02
GCNT4	GCNT4	1	0	-3.2	6.63E-02
CDK17	CDK17	1	0	-3.2	6.05E-02
CBL	CBL	1	1	-3.2	1.71E-02
UTRN	UTRN	1	1	-3.2	1.92E-02
IL6ST	IL6ST	0	1	-3.3	1.17E-02
SSH2	SSH2	0	1	-3.8	2.38E-02
RAPGEF6	RAPGEF6	1	0	-4.1	1.20E-02
KMT2A	KMT2A	0	1	-5.5	2.00E-02

a FC from NR datbase is used for reporting unless not available replaced by Ensembl database

Table S4_DGEs among pigs from different treatment groups at baseline

Ensembl Database

Gene ID Ensembl	Gene ID	FC	padj	Annotation
ENSSSCG00000000194	TUBA1C	-3.06258	1.46E-07	Tubulin alpha-1C chain; Tubulin is the major constituent of microtubules. It binds two moles of GTP, one at an exchangeable site on the beta chain and one at a non-exchangeable site on the alpha chain; Belongs to the tubulin family
ENSSSCG00000037524	LY6D	-4.04719	8.98E-06	Lymphocyte antigen 6D; May act as a specification marker at earliest stage specification of lymphocytes between B- and T-cell development. Marks the earliest stage of B-cell specification; LY6/PLAUR domain containing
ENSSSCG00000017307	MYL4	-4.98969	1.34E-04	Myosin light chain 4; Regulatory light chain of myosin. Does not bind calcium; EF-hand domain containing
ENSSSCG00000035226	SSCG0000003	5.462148	6.32E-03	Pig gene unknown function, no orthologues with Primates

NR database

Gene ID	FC	padj	Annotation
STXBP1_tv1	-3.8583	1.42E-02	This gene encodes a syntaxin-binding protein. The encoded protein appears to play a role in release of neurotransmitters via regulation of syntaxin, a transmembrane attachment protein receptor.

Table S5. Real time PCR validation of DGEs in whole blood of pigs fed FV-supplemented or Control diet after two weeks

				Time		Diet (d 14)	
**GENE**	**Diet**	**Baseline**	**Day 14**	**adj p-value**	**FC**	**adj p-value**	**FC**
IL7R	Control	4.67 ± 0.29 *	4.31 ± 0.11	0.3107		**0.0069**	-1.6
	FV	4.64 ± 0.07	5.02 ± 0.09	**0.0301**	-1.3		
TNFRSF13	Control	10.53 ± 0.21	9.99 ± 0.16	0.0943		**0.0345**	1.6
	FV	10.05 ± 0.25	9.28 ± 0.18	0.0674			
LY86	Control	6.57 ± 0.42	5.74 ± 0.16	0.1145			
	FV	6.86 ± 0.03	5.42 ± 0.37	**0.018**	2.7		
IL1R2	Control	6.15 ± 0.77	7.01 ± 0.26	0.336			
	FV	4.97 ± 0.17	7.15 ± 0.06	**0.0003**	-4.5		
CEBPB	Control	5.72 ± 0.52	6.83 ± 0.25	0.1014			
	FV	5.18 ± 0.10	6.95 ± 0.17	**0.001**	-3.4		

* values represent mean ± SE of adjusted CT values for each gene. DGE is denoted with p-value when comparions among time (D14 vs D0) or dietary treatment (FV vs C) at day 14 (data not shown).. No significance was detected for other genes: SLA-DRA, SLA-DQA,CD 79A or IL6ST (data not shown)

Table S6_Top Biological functions predicted by Ingenuity Pathway analysis

Categories	Diseases or Functions Annotation	p-value	Activation z-score	Molecules	# Molecules
Cellular Movement	Migration of cells	6.08E-06	-2.50	*ANGPTL4,BCL6,BTK,C5AR1,CBL,CD72,CD74,CSF2RB, CXCR4,CYBB,DNMT1,DOCK8,DUSP22,EIF2AK4,ENG, ERAP1,F12,FAM89B,FN1,FOXO3,HSPB1,ID3,IFNAR1, IGHA1,IL6ST,IL7R,ILK,IQGAP1,ITGA4,ITGAL,ITGB1, JAK1,JCHAIN,KLF2,KMT2A,LDLR,LGALS1,LRRK2,LYST, MECP2,MIF,MT-ND1 ,MYH9, NFATC3,NLRP3, PELI1, PER1, PIK3IP1, PLXNC1, POU2AF1, PREX1,PTPRC, RPL13A,S100A10, S100A4,SGK1, SIGIRR,SSH2, TGFBI, THBS1, THY1, TUBB2B,USP9X,VAV2,VCAN, WIPF1, XBP1*	67
Inflammatory Response	Immune response of cells	1.17E-04	-2.59	*BAK1,BCL2L1,C5AR1,CD79A,CSF2RB,DOCK8,FN1,HSPB 1,IFNAR1,IGHA1,IL7R,ITGA4,ITGAL,ITGB1,JAK1,NCOA2, NLRP3,THBS1*	18
Cell-To-Cell Signaling and Interaction	Binding of lymphatic system cells	1.22E-05	-3.24	*BTK,CSF2RB,CXCR4,ENG,FN1,IL6ST,IL7R,ITGA4,ITGAL,I TGB1,JAK1,MSN,NFATC3,THBS1*	14
Cell-To-Cell Signaling and Interaction,Hematological System Development and Function	Binding of mononuclear leukocytes	4.23E-05	-2.69	*BTK,CXCR4,ENG,FN1,IL6ST,IL7R,ITGA4,ITGAL,ITGB1,JA K1,MIF,MSN,NFATC3,THBS1*	14
Cell-To-Cell Signaling and Interaction,Hematological System Development and Function	Binding of lymphocytes	2.47E-05	-3.09	*BTK,CXCR4,ENG,FN1,IL6ST,IL7R,ITGA4,ITGAL,ITGB1,JA K1,MSN,NFATC3,THBS1*	13
Cell Death and Survival,Cellular Compromise	Cytotoxicity of leukocytes	7.28E-04	-2.56	*CYBB,DOCK8,FN1,HLA-DRA,IFNAR1,ITGAL, LDLR,MYH9,NOTCH2,PTPRC,WIPF1*	11
Cell-To-Cell Signaling and Interaction,Hematological System Development and Function,Immune Cell Trafficking	Adhesion of lymphocytes	1.11E-04	-2.77	*BTK,CXCR4,FN1,IL6ST,IL7R,ITGA4,ITGAL,ITGB1,JAK1,T HBS1*	10
Hematological System Development and Function,Lymphoid Tissue Structure and Development,Tissue Morphology	Quantity of natural killer T lymphocytes	2.05E-05	-2.55	*CSF2RB,ID3,IL7R,ITGAL,KMT2E,NOTCH2,PTPRC,TNFAI P3,TRAF2,TXNIP*	10

Z-scores per each biological function affected with contributing genes. The Z-score algorithm is designed to reduce the chance that random data will generate significant predictions. Negative Z-scores indicate a down-regulation of biofunction, positive Z-scores indicate an up-regulation of function. Absolute Z scores values >2.0 are recommended to be used for biological predictions

Table S7. Overlap of joint NR & WG DEGs and GSEA database

**Collection(s):**	C7
# overlaps shown:	10
# genesets in collections:	5219
# genes in comparison (n):	325
# genes in universe (N):	40312

Gene Set Name	# Genes in	Description	# Genes in Overlap (	k/K	p-value	FDR q-value
HARALAMBIEVA_PBMC_FLUARIX_AGE_50_74YO_CORR_WITH_28D_MEM_B_CELL_RESPONSE_AT_28DY_POSITIVE	1249	Genes positively correlated with memory B cell response at 28d in peripheral blood mononuclear cell in seniors (50-74) after exposure to Fluarix , time point 28D	60	0.048	5.36E-29	2.80E-25
GSE26488_CTRL_VS_PEPTIDE_INJECTION_OT2_THYMOCYTE_DN	183	Genes down-regulated in double positive thymocytes from OT-2 transgenic mice: control versus injected with agonist peptide.	25	0.1366	2.42E-23	6.32E-20
OSMAN_BLOOD_CHAD63_KH_AGE_18_50YO_HIGH_DOSE_SUBJECTS_24HR_UP	1992	Genes up-regulated in blood 24hr vs 0hr in adults (18-50) (high dose subjects) after exposure to ChAd63-KH , time point 24H , administered Intramuscular injection. Comment: DE gene list for high dose subjects.	65	0.0326	3.75E-22	6.53E-19
ZAK_PBMC_MRKAD5_HIV_1_GAG_POL_NEF_AGE_20_50YO_1DY_UP	1012	Genes up-regulated in peripheral blood mononuclear cell 1d vs 0d in adults (20-50) after exposure to MRKAd5 HIV-1 gag/pol/nef , time point 1D. Comment: Table includes specific cell types	43	0.0425	7.14E-19	9.32E-16
GSE3720_LPS_VS_PMA_STIM_VD1_GAMMADELTA_TCELL_DN	172	Genes down-regulated in Vd1 gamma delta T cells: LPS versus phorbol myristate acetate and ionomycin [PubChem=4792;3733].	21	0.1221	9.16E-19	9.56E-16
GSE25677_MPL_VS_R848_STIM_BCELL_DN	181	Genes down-regulated in B lymphocytes after immunization with: monophosphoryl lipid A versus imiquimod [PubChem=13982876].	21	0.116	2.67E-18	2.33E-15
NAKAYA_PBMC_FLUMIST_AGE_18_50YO_3DY_DN	680	Genes down-regulated in peripheral blood mononuclear cell 3d vs 0d in adults (18-50) after exposure to FluMist , time point 3D. Comment: Supplementary Table 1b: All the differentially expressed genes identified in PBMCs of TIV vaccinees.	35	0.0515	4.62E-18	3.45E-15
GSE3720_UNSTIM_VS_LPS_STIM_VD2_GAMMADELTA_TCELL_UP	167	Genes up-regulated in Vd2 gamma delta T cells: untreated versus LPS.	20	0.1198	9.19E-18	6.00E-15
GSE29618_PDC_VS_MDC_DN	200	Genes down-regulated in comparison of plasmacytoid dendritic cells (DC) versus myeloid DCs.	21	0.105	2.14E-17	1.24E-14
ZAK_PBMC_MRKAD5_HIV_1_GAG_POL_NEF_AGE_20_50YO_1DY_DN	1010	Genes down-regulated in peripheral blood mononuclear cell 1d vs 0d in adults (20-50) after exposure to MRKAd5 HIV-1 gag/pol/nef , time point 1D. Comment: Table includes specific cell types	40	0.0396	1.42E-16	7.44E-14

Gene/Gene Set Overlap Matrix

Entrez Gene Id	Gene Symbol	Gene Description	HARALAMBIEVA_PBMC_FLUARIX_AGE_50_74YO_CORR_WITH_28D_MEM_B_CELL_RESPONSE_AT_28DY_POSITIVE	GSE26488_CTRL_VS_PEPTIDE_INJECTION_OT2_THYMOCYTE_DN	OSMAN_BLOOD_CHAD63_KH_AGE_18_50YO_HIGH_DOSE_SUBJECTS_24HR_UP	ZAK_PBMC_MRKAD5_HIV_1_GAG_POL_NEF_AGE_20_50YO_1DY_UP	GSE3720_LPS_VS_PMA_STIM_VD1_GAMMADELTA_TCELL_DN	GSE25677_MPL_VS_R848_STIM_BCELL_DN	NAKAYA_PBMC_FLUMIST_AGE_18_50YO_3DY_DN	GSE3720_UNSTIM_VS_LPS_STIM_VD2_GAMMADELTA_TCELL_UP	GSE29618_PDC_VS_MDC_DN	ZAK_PBMC_MRKAD5_HIV_1_GAG_POL_NEF_AGE_20_50YO_1DY_DN
604	BCL6	BCL6 transcription repressor [Source:HGNC Symbol;Acc:HGNC:1001]	X	X		X		X			X	
51363	CHST15	carbohydrate sulfotransferase 15 [Source:HGNC Symbol;Acc:HGNC:18137]	X	X		X		X				
8301	PICALM	phosphatidylinositol binding clathrin assembly protein [Source:HGNC Symbol;Acc:HGNC:15514]	X	X				X			X	
6446	SGK1	serum/glucocorticoid regulated kinase 1 [Source:HGNC Symbol;Acc:HGNC:10810]	X	X							X	
10365	KLF2	Kruppel like factor 2 [Source:HGNC Symbol;Acc:HGNC:6347]	X	X								
115650	TNFRSF13C	TNF receptor superfamily member 13C [Source:HGNC Symbol;Acc:HGNC:17755]	X	X								
55603	TENT5A	terminal nucleotidyltransferase 5A [Source:HGNC Symbol;Acc:HGNC:18345]	X		X	X		X		X		
1051	CEBPB	CCAAT enhancer binding protein beta [Source:HGNC Symbol;Acc:HGNC:1834]	X		X	X			X			
7150	TOP1	DNA topoisomerase I [Source:HGNC Symbol;Acc:HGNC:11986]	X		X	X			X			
3122	HLA-DRA	major histocompatibility complex, class II, DR alpha [Source:HGNC Symbol;Acc:HGNC:4947]	X		X	X					X	
114548	NLRP3	NLR family pyrin domain containing 3 [Source:HGNC Symbol;Acc:HGNC:16400]	X		X	X						
57162	PELI1	pellino E3 ubiquitin protein ligase 1 [Source:HGNC Symbol;Acc:HGNC:8827]	X		X	X						
1439	CSF2RB	colony stimulating factor 2 receptor subunit beta [Source:HGNC Symbol;Acc:HGNC:2436]	X		X	X						
221472	FGD2	FYVE, RhoGEF and PH domain containing 2 [Source:HGNC Symbol;Acc:HGNC:3664]	X		X	X						
695	BTK	Bruton tyrosine kinase [Source:HGNC Symbol;Acc:HGNC:1133]	X		X	X						
1536	CYBB	cytochrome b-245 beta chain [Source:HGNC Symbol;Acc:HGNC:2578]	X		X	X						
2180	ACSL1	acyl-CoA synthetase long chain family member 1 [Source:HGNC Symbol;Acc:HGNC:3569]	X		X	X						
4084	MXD1	MAX dimerization protein 1 [Source:HGNC Symbol;Acc:HGNC:6761]	X		X	X						
3433	IFIT2	interferon induced protein with tetratricopeptide repeats 2 [Source:HGNC Symbol;Acc:HGNC:5409]	X		X	X						
3759	KCNJ2	potassium inwardly rectifying channel subfamily J member 2 [Source:HGNC Symbol;Acc:HGNC:6263]	X		X	X						
3949	LDLR	low density lipoprotein receptor [Source:HGNC Symbol;Acc:HGNC:6547]	X		X	X						
120892	LRRK2	leucine rich repeat kinase 2 [Source:HGNC Symbol;Acc:HGNC:18618]	X		X	X						
2182	ACSL4	acyl-CoA synthetase long chain family member 4 [Source:HGNC Symbol;Acc:HGNC:3571]	X		X	X						
578	BAK1	BCL2 antagonist/killer 1 [Source:HGNC Symbol;Acc:HGNC:949]	X		X	X						
3727	JUND	JunD proto-oncogene, AP-1 transcription factor subunit [Source:HGNC Symbol;Acc:HGNC:6206]	X		X				X			

221037	JMJD1C	jumonji domain containing 1C [Source:HGNC Symbol;Acc:HGNC:12313]	X		X				X			
3964	LGALS8	galectin 8 [Source:HGNC Symbol;Acc:HGNC:6569]	X		X							
1537	CYC1	cytochrome c1 [Source:HGNC Symbol;Acc:HGNC:2579]	X		X							
1130	LYST	lysosomal trafficking regulator [Source:HGNC Symbol;Acc:HGNC:1968]	X			X	X			X		
10154	PLXNC1	plexin C1 [Source:HGNC Symbol;Acc:HGNC:9106]	X			X					X	
10221	TRIB1	tribbles pseudokinase 1 [Source:HGNC Symbol;Acc:HGNC:16891]	X			X					X	
728	C5AR1	complement C5a receptor 1 [Source:HGNC Symbol;Acc:HGNC:1338]	X			X						
57178	ZMIZ1	zinc finger MIZ-type containing 1 [Source:HGNC Symbol;Acc:HGNC:16493]	X			X						
81553	CYRIA	CYFIP related Rac1 interactor A [Source:HGNC Symbol;Acc:HGNC:25373]	X			X						
84669	USP32	ubiquitin specific peptidase 32 [Source:HGNC Symbol;Acc:HGNC:19143]	X			X						
1774	DNASE1L1	deoxyribonuclease 1 like 1 [Source:HGNC Symbol;Acc:HGNC:2957]	X			X						
4793	NFKBIB	NFKB inhibitor beta [Source:HGNC Symbol;Acc:HGNC:7798]	X				X		X			
2309	FOXO3	forkhead box O3 [Source:HGNC Symbol;Acc:HGNC:3821]	X						X			
64795	RMND5A	required for meiotic nuclear division 5 homolog A [Source:HGNC Symbol;Acc:HGNC:25850]	X						X			
509	ATP5F1C	ATP synthase F1 subunit gamma [Source:HGNC Symbol;Acc:HGNC:833]	X							X		
9450	LY86	lymphocyte antigen 86 [Source:HGNC Symbol;Acc:HGNC:16837]	X								X	
2950	GSTP1	glutathione S-transferase pi 1 [Source:HGNC Symbol;Acc:HGNC:4638]	X								X	
8876	VNN1	vanin 1 [Source:HGNC Symbol;Acc:HGNC:12705]	X									X
10788	IQGAP2	IQ motif containing GTPase activating protein 2 [Source:HGNC Symbol;Acc:HGNC:6111]	X									
83716	CRISPLD2	cysteine rich secretory protein LCCL domain containing 2 [Source:HGNC Symbol;Acc:HGNC:25248]	X									
23389	MED13L	mediator complex subunit 13L [Source:HGNC Symbol;Acc:HGNC:22962]	X									
7045	TGFBI	transforming growth factor beta induced [Source:HGNC Symbol;Acc:HGNC:11771]	X									
5452	POU2F2	POU class 2 homeobox 2 [Source:HGNC Symbol;Acc:HGNC:9213]	X									
9236	CCPG1	cell cycle progression 1 [Source:HGNC Symbol;Acc:HGNC:24227]	X									
80315	CPEB4	cytoplasmic polyadenylation element binding protein 4 [Source:HGNC Symbol;Acc:HGNC:21747]	X									
7410	VAV2	vav guanine nucleotide exchange factor 2 [Source:HGNC Symbol;Acc:HGNC:12658]	X									
1978	EIF4EBP1	eukaryotic translation initiation factor 4E binding protein 1 [Source:HGNC Symbol;Acc:HGNC:3288]	X									
29924	EPN1	epsin 1 [Source:HGNC Symbol;Acc:HGNC:21604]	X									
533	ATP6V0B	ATPase H+ transporting V0 subunit b [Source:HGNC Symbol;Acc:HGNC:861]	X									
91947	ARRDC4	arrestin domain containing 4 [Source:HGNC Symbol;Acc:HGNC:28087]	X									
10381	TUBB3	tubulin beta 3 class III [Source:HGNC Symbol;Acc:HGNC:20772]	X									
91748	MIDEAS	mitotic deacetylase associated SANT domain protein [Source:HGNC Symbol;Acc:HGNC:19853]	X									
8031	NCOA4	nuclear receptor coactivator 4 [Source:HGNC Symbol;Acc:HGNC:7671]	X									
8482	SEMA7A	semaphorin 7A (John Milton Hagen blood group) [Source:HGNC Symbol;Acc:HGNC:10741]	X									
83460	EMC6	ER membrane protein complex subunit 6 [Source:HGNC Symbol;Acc:HGNC:28430]	X									
2014	EMP3	epithelial membrane protein 3 [Source:HGNC Symbol;Acc:HGNC:3335]		X	X	X					X	
9969	MED13	mediator complex subunit 13 [Source:HGNC Symbol;Acc:HGNC:22474]		X	X			X	X			
9535	GMFG	glia maturation factor gamma [Source:HGNC Symbol;Acc:HGNC:4374]		X			X	X		X	X	
6304	SATB1	SATB homeobox 1 [Source:HGNC Symbol;Acc:HGNC:10541]		X			X	X		X		X
51735	RAPGEF6	Rap guanine nucleotide exchange factor 6 [Source:HGNC Symbol;Acc:HGNC:20655]		X			X					X
6653	SORL1	sortilin related receptor 1 [Source:HGNC Symbol;Acc:HGNC:11185]		X				X	X			X
54014	BRWD1	bromodomain and WD repeat domain containing 1 [Source:HGNC Symbol;Acc:HGNC:12760]		X				X	X			X
3716	JAK1	Janus kinase 1 [Source:HGNC Symbol;Acc:HGNC:6190]		X				X	X			
6281	S100A10	S100 calcium binding protein A10 [Source:HGNC Symbol;Acc:HGNC:10487]		X				X			X	
4478	MSN	moesin [Source:HGNC Symbol;Acc:HGNC:7373]		X				X			X	
8826	IQGAP1	IQ motif containing GTPase activating protein 1 [Source:HGNC Symbol;Acc:HGNC:6110]		X				X			X	
28951	TRIB2	tribbles pseudokinase 2 [Source:HGNC Symbol;Acc:HGNC:30809]		X				X				X
10129	FRY	FRY microtubule binding protein [Source:HGNC Symbol;Acc:HGNC:20367]		X				X				
4627	MYH9	myosin heavy chain 9 [Source:HGNC Symbol;Acc:HGNC:7579]		X				X				
23043	TNIK	TRAF2 and NCK interacting kinase [Source:HGNC Symbol;Acc:HGNC:30765]		X					X			X
7852	CXCR4	C-X-C motif chemokine receptor 4 [Source:HGNC Symbol;Acc:HGNC:2561]		X								X
256380	SCML4	Scm polycomb group protein like 4 [Source:HGNC Symbol;Acc:HGNC:21397]		X								X
3399	ID3	inhibitor of DNA binding 3, HLH protein [Source:HGNC Symbol;Acc:HGNC:5362]		X								
4204	MECP2	methyl-CpG binding protein 2 [Source:HGNC Symbol;Acc:HGNC:6990]		X								
4061	LY6E	lymphocyte antigen 6 family member E [Source:HGNC Symbol;Acc:HGNC:6727]			X	X	X			X		
5720	PSME1	proteasome activator subunit 1 [Source:HGNC Symbol;Acc:HGNC:9568]			X	X	X					
51316	PLAC8	placenta associated 8 [Source:HGNC Symbol;Acc:HGNC:19254]			X	X				X		
6050	RNH1	ribonuclease/angiogenin inhibitor 1 [Source:HGNC Symbol;Acc:HGNC:10074]			X	X					X	
3434	IFIT1	interferon induced protein with tetratricopeptide repeats 1 [Source:HGNC Symbol;Acc:HGNC:5407]			X	X						
23001	WDFY3	WD repeat and FYVE domain containing 3 [Source:HGNC Symbol;Acc:HGNC:20751]			X	X						
79670	TUT7	terminal uridylyl transferase 7 [Source:HGNC Symbol;Acc:HGNC:25817]			X	X						
7402	UTRN	utrophin [Source:HGNC Symbol;Acc:HGNC:12635]			X	X						
80124	VCPIP1	valosin containing protein interacting protein 1 [Source:HGNC Symbol;Acc:HGNC:30897]			X	X						
84641	MFSD14B	major facilitator superfamily domain containing 14B [Source:HGNC Symbol;Acc:HGNC:23376]			X	X						
9334	B4GALT5	beta-1,4-galactosyltransferase 5 [Source:HGNC Symbol;Acc:HGNC:928]			X	X						
9246	UBE2L6	ubiquitin conjugating enzyme E2 L6 [Source:HGNC Symbol;Acc:HGNC:12490]			X	X						
7128	TNFAIP3	TNF alpha induced protein 3 [Source:HGNC Symbol;Acc:HGNC:11896]			X				X			
57018	CCNL1	cyclin L1 [Source:HGNC Symbol;Acc:HGNC:20569]			X				X			
5128	CDK17	cyclin dependent kinase 17 [Source:HGNC Symbol;Acc:HGNC:8750]			X				X			
8672	EIF4G3	eukaryotic translation initiation factor 4 gamma 3 [Source:HGNC Symbol;Acc:HGNC:3298]			X				X			
55183	RIF1	replication timing regulatory factor 1 [Source:HGNC Symbol;Acc:HGNC:23207]			X				X			

3956	LGALS1	galectin 1 [Source:HGNC Symbol;Acc:HGNC:6561]	X						X	
5788	PTPRC	protein tyrosine phosphatase receptor type C [Source:HGNC Symbol;Acc:HGNC:9666]	X						X	
3597	IL13RA1	interleukin 13 receptor subunit alpha 1 [Source:HGNC Symbol;Acc:HGNC:5974]	X						X	
3097	HIVEP2	HIVEP zinc finger 2 [Source:HGNC Symbol;Acc:HGNC:4921]	X							X
10628	TXNIP	thioredoxin interacting protein [Source:HGNC Symbol;Acc:HGNC:16952]	X							
3611	ILK	integrin linked kinase [Source:HGNC Symbol;Acc:HGNC:6040]	X							
6158	RPL28	ribosomal protein L28 [Source:HGNC Symbol;Acc:HGNC:10330]	X							
387751	GVINP1	GTPase, very large interferon inducible pseudogene 1 [Source:HGNC Symbol;Acc:HGNC:25813]	X							
11214	AKAP13	A-kinase anchoring protein 13 [Source:HGNC Symbol;Acc:HGNC:371]	X							
113	ADCY7	adenylate cyclase 7 [Source:HGNC Symbol;Acc:HGNC:238]	X							
3676	ITGA4	integrin subunit alpha 4 [Source:HGNC Symbol;Acc:HGNC:6140]	X							
51752	ERAP1	endoplasmic reticulum aminopeptidase 1 [Source:HGNC Symbol;Acc:HGNC:18173]	X							
47	ACLY	ATP citrate lyase [Source:HGNC Symbol;Acc:HGNC:115]	X							
972	CD74	CD74 molecule [Source:HGNC Symbol;Acc:HGNC:1697]	X							
84162	KIAA1109	KIAA1109 [Source:HGNC Symbol;Acc:HGNC:26953]	X							
10499	NCOA2	nuclear receptor coactivator 2 [Source:HGNC Symbol;Acc:HGNC:7669]	X							
10287	RGS19	regulator of G protein signaling 19 [Source:HGNC Symbol;Acc:HGNC:13735]	X							
81704	DOCK8	dedicator of cytokinesis 8 [Source:HGNC Symbol;Acc:HGNC:19191]	X							
10095	ARPC1B	actin related protein 2/3 complex subunit 1B [Source:HGNC Symbol;Acc:HGNC:704]	X							
23304	UBR2	ubiquitin protein ligase E3 component n-recognin 2 [Source:HGNC Symbol;Acc:HGNC:21289]	X							
53840	TRIM34	tripartite motif containing 34 [Source:HGNC Symbol;Acc:HGNC:10063]	X							
64167	ERAP2	endoplasmic reticulum aminopeptidase 2 [Source:HGNC Symbol;Acc:HGNC:29499]	X							
27338	UBE2S	ubiquitin conjugating enzyme E2 S [Source:HGNC Symbol;Acc:HGNC:17895]	X							
1984	EIF5A	eukaryotic translation initiation factor 5A [Source:HGNC Symbol;Acc:HGNC:3300]	X							
4853	NOTCH2	notch receptor 2 [Source:HGNC Symbol;Acc:HGNC:7882]		X						
79895	ATP8B4	ATPase phospholipid transporting 8B4 (putative) [Source:HGNC Symbol;Acc:HGNC:13536]		X						
29997	NOP53	NOP53 ribosome biogenesis factor [Source:HGNC Symbol;Acc:HGNC:4333]			X			X		X
6141	RPL18	ribosomal protein L18 [Source:HGNC Symbol;Acc:HGNC:10310]			X			X		X
4050	LTB	lymphotoxin beta [Source:HGNC Symbol;Acc:HGNC:6711]			X			X		X
6208	RPS14	ribosomal protein S14 [Source:HGNC Symbol;Acc:HGNC:10387]			X					X
51621	KLF13	Kruppel like factor 13 [Source:HGNC Symbol;Acc:HGNC:13672]			X					
84153	RNASEH2C	ribonuclease H2 subunit C [Source:HGNC Symbol;Acc:HGNC:24116]			X					
23091	ZC3H13	zinc finger CCCH-type containing 13 [Source:HGNC Symbol;Acc:HGNC:20368]			X					
8666	EIF3G	eukaryotic translation initiation factor 3 subunit G [Source:HGNC Symbol;Acc:HGNC:3274]			X					
4282	MIF	macrophage migration inhibitory factor [Source:HGNC Symbol;Acc:HGNC:7097]			X					
259230	SGMS1	sphingomyelin synthase 1 [Source:HGNC Symbol;Acc:HGNC:29799]			X					
25796	PGLS	6-phosphogluconolactonase [Source:HGNC Symbol;Acc:HGNC:8903]			X					
79144	PPDPF	pancreatic progenitor cell differentiation and proliferation factor [Source:HGNC Symbol;Acc:HGNC:16142]			X					
54842	MFSD6	major facilitator superfamily domain containing 6 [Source:HGNC Symbol;Acc:HGNC:24711]			X					
90326	THAP3	THAP domain containing 3 [Source:HGNC Symbol;Acc:HGNC:20855]			X					
27125	AFF4	AF4/FMR2 family member 4 [Source:HGNC Symbol;Acc:HGNC:17869]				X	X			
1786	DNMT1	DNA methyltransferase 1 [Source:HGNC Symbol;Acc:HGNC:2976]				X				
55904	KMT2E	lysine methyltransferase 2E (inactive) [Source:HGNC Symbol;Acc:HGNC:18541]				X				
85464	SSH2	slingshot protein phosphatase 2 [Source:HGNC Symbol;Acc:HGNC:30580]				X				
3454	IFNAR1	interferon alpha and beta receptor subunit 1 [Source:HGNC Symbol;Acc:HGNC:5432]				X				
5922	RASA2	RAS p21 protein activator 2 [Source:HGNC Symbol;Acc:HGNC:9872]					X			X
5583	PRKCH	protein kinase C eta [Source:HGNC Symbol;Acc:HGNC:9403]					X			X
25909	AHCTF1	AT-hook containing transcription factor 1 [Source:HGNC Symbol;Acc:HGNC:24618]					X			X
5187	PER1	period circadian regulator 1 [Source:HGNC Symbol;Acc:HGNC:8845]					X			
1195	CLK1	CDC like kinase 1 [Source:HGNC Symbol;Acc:HGNC:2068]					X			
204851	HIPK1	homeodomain interacting protein kinase 1 [Source:HGNC Symbol;Acc:HGNC:19006]					X			
26058	GIGYF2	GRB10 interacting GYF protein 2 [Source:HGNC Symbol;Acc:HGNC:11960]					X			
7057	THBS1	thrombospondin 1 [Source:HGNC Symbol;Acc:HGNC:11785]					X			
7320	UBE2B	ubiquitin conjugating enzyme E2 B [Source:HGNC Symbol;Acc:HGNC:12473]					X			
22850	ADNP2	ADNP homeobox 2 [Source:HGNC Symbol;Acc:HGNC:23803]					X			
2033	EP300	E1A binding protein p300 [Source:HGNC Symbol;Acc:HGNC:3373]					X			
7456	WIPF1	WAS/WASL interacting protein family member 1 [Source:HGNC Symbol;Acc:HGNC:12736]					X			
56261	GPCPD1	glycerophosphocholine phosphodiesterase 1 [Source:HGNC Symbol;Acc:HGNC:26957]					X			
10625	IVNS1ABP	influenza virus NS1A binding protein [Source:HGNC Symbol;Acc:HGNC:16951]					X			
9728	SECISBP2L	SECIS binding protein 2 like [Source:HGNC Symbol;Acc:HGNC:28997]					X			
8916	HERC3	HECT and RLD domain containing E3 ubiquitin protein ligase 3 [Source:HGNC Symbol;Acc:HGNC:4876]					X			
8079	MLF2	myeloid leukemia factor 2 [Source:HGNC Symbol;Acc:HGNC:7126]					X			
8665	EIF3F	eukaryotic translation initiation factor 3 subunit F [Source:HGNC Symbol;Acc:HGNC:3275]						X		X
6132	RPL8	ribosomal protein L8 [Source:HGNC Symbol;Acc:HGNC:10368]						X		X
6161	RPL32	ribosomal protein L32 [Source:HGNC Symbol;Acc:HGNC:10336]						X		X
3575	IL7R	interleukin 7 receptor [Source:HGNC Symbol;Acc:HGNC:6024]						X		X
6224	RPS20	ribosomal protein S20 [Source:HGNC Symbol;Acc:HGNC:10405]						X		X
6203	RPS9	ribosomal protein S9 [Source:HGNC Symbol;Acc:HGNC:10442]						X		
971	CD72	CD72 molecule [Source:HGNC Symbol;Acc:HGNC:1696]						X		
340152	ZC3H12D	zinc finger CCCH-type containing 12D [Source:HGNC Symbol;Acc:HGNC:21175]						X		
4715	NDUFB9	NADH:ubiquinone oxidoreductase subunit B9 [Source:HGNC Symbol;Acc:HGNC:7704]						X		

51491	NOP16	NOP16 nucleolar protein [Source:HGNC Symbol;Acc:HGNC:26934]	X		
256987	SERINC5	serine incorporator 5 [Source:HGNC Symbol;Acc:HGNC:18825]		X	X
6275	S100A4	S100 calcium binding protein A4 [Source:HGNC Symbol;Acc:HGNC:10494]		X	
1955	MEGF9	multiple EGF like domains 9 [Source:HGNC Symbol;Acc:HGNC:3234]		X	
909	CD1A	CD1a molecule [Source:HGNC Symbol;Acc:HGNC:1634]		X	
9736	USP34	ubiquitin specific peptidase 34 [Source:HGNC Symbol;Acc:HGNC:20066]			X
113791	PIK3IP1	phosphoinositide-3-kinase interacting protein 1 [Source:HGNC Symbol;Acc:HGNC:24942]			X
11224	RPL35	ribosomal protein L35 [Source:HGNC Symbol;Acc:HGNC:10344]			X
6193	RPS5	ribosomal protein S5 [Source:HGNC Symbol;Acc:HGNC:10426]			X
4297	KMT2A	lysine methyltransferase 2A [Source:HGNC Symbol;Acc:HGNC:7132]			X
4775	NFATC3	nuclear factor of activated T cells 3 [Source:HGNC Symbol;Acc:HGNC:7777]			X
3007	H1-3	H1.3 linker histone, cluster member [Source:HGNC Symbol;Acc:HGNC:4717]			X
973	CD79A	CD79a molecule [Source:HGNC Symbol;Acc:HGNC:1698]			X
974	CD79B	CD79b molecule [Source:HGNC Symbol;Acc:HGNC:1699]			X
3495	IGHD	immunoglobulin heavy constant delta [Source:HGNC Symbol;Acc:HGNC:5480]			X
931	MS4A1	membrane spanning 4-domains A1 [Source:HGNC Symbol;Acc:HGNC:7315]			X
640	BLK	BLK proto-oncogene, Src family tyrosine kinase [Source:HGNC Symbol;Acc:HGNC:1057]			X
23195	MDN1	midasin AAA ATPase 1 [Source:HGNC Symbol;Acc:HGNC:18302]			X
23345	SYNE1	spectrin repeat containing nuclear envelope protein 1 [Source:HGNC Symbol;Acc:HGNC:17089]			X
23394	ADNP	activity dependent neuroprotector homeobox [Source:HGNC Symbol;Acc:HGNC:15766]			X
51301	GCNT4	glucosaminyl (N-acetyl) transferase 4 [Source:HGNC Symbol;Acc:HGNC:17973]			X
28526	TRDC	T cell receptor delta constant [Source:HGNC Symbol;Acc:HGNC:12253]			X
59307	SIGIRR	single Ig and TIR domain containing [Source:HGNC Symbol;Acc:HGNC:30575]			
1936	EEF1D	eukaryotic translation elongation factor 1 delta [Source:HGNC Symbol;Acc:HGNC:3211]			
1462	VCAN	versican [Source:HGNC Symbol;Acc:HGNC:2464]			
3683	ITGAL	integrin subunit alpha L [Source:HGNC Symbol;Acc:HGNC:6148]			
6840	SVIL	supervillin [Source:HGNC Symbol;Acc:HGNC:11480]			
7850	IL1R2	interleukin 1 receptor type 2 [Source:HGNC Symbol;Acc:HGNC:5994]			
6130	RPL7A	ribosomal protein L7a [Source:HGNC Symbol;Acc:HGNC:10364]			
8407	TAGLN2	transgelin 2 [Source:HGNC Symbol;Acc:HGNC:11554]			
6134	RPL10	ribosomal protein L10 [Source:HGNC Symbol;Acc:HGNC:10298]			
1396	CRIP1	cysteine rich protein 1 [Source:HGNC Symbol;Acc:HGNC:2360]			
9398	CD101	CD101 molecule [Source:HGNC Symbol;Acc:HGNC:5949]			
7764	ZNF217	zinc finger protein 217 [Source:HGNC Symbol;Acc:HGNC:13009]			
1362	CPD	carboxypeptidase D [Source:HGNC Symbol;Acc:HGNC:2301]			
23521	RPL13A	ribosomal protein L13a [Source:HGNC Symbol;Acc:HGNC:10304]			
7328	UBE2H	ubiquitin conjugating enzyme E2 H [Source:HGNC Symbol;Acc:HGNC:12484]			
25824	PRDX5	peroxiredoxin 5 [Source:HGNC Symbol;Acc:HGNC:9355]			
7053	TGM3	transglutaminase 3 [Source:HGNC Symbol;Acc:HGNC:11779]			
56940	DUSP22	dual specificity phosphatase 22 [Source:HGNC Symbol;Acc:HGNC:16077]			
283131	NEAT1	nuclear paraspeckle assembly transcript 1 [Source:HGNC Symbol;Acc:HGNC:30815]			
10263	CDK2AP2	cyclin dependent kinase 2 associated protein 2 [Source:HGNC Symbol;Acc:HGNC:30833]			
22877	MLXIP	MLX interacting protein [Source:HGNC Symbol;Acc:HGNC:17055]			
55252	ASXL2	ASXL transcriptional regulator 2 [Source:HGNC Symbol;Acc:HGNC:23805]			
4012	LNPEP	leucyl and cystinyl aminopeptidase [Source:HGNC Symbol;Acc:HGNC:6656]			
54491	OTULINL	OTU deubiquitinase with linear linkage specificity like [Source:HGNC Symbol;Acc:HGNC:25629]			
23054	NCOA6	nuclear receptor coactivator 6 [Source:HGNC Symbol;Acc:HGNC:15936]			
6670	SP3	Sp3 transcription factor [Source:HGNC Symbol;Acc:HGNC:11208]			
9698	PUM1	pumilio RNA binding family member 1 [Source:HGNC Symbol;Acc:HGNC:14957]			
2289	FKBP5	FKBP prolyl isomerase 5 [Source:HGNC Symbol;Acc:HGNC:3721]			
79901	CYBRD1	cytochrome b reductase 1 [Source:HGNC Symbol;Acc:HGNC:20797]			
57448	BIRC6	baculoviral IAP repeat containing 6 [Source:HGNC Symbol;Acc:HGNC:13516]			
8673	VAMP8	vesicle associated membrane protein 8 [Source:HGNC Symbol;Acc:HGNC:12647]			
55291	PPP6R3	protein phosphatase 6 regulatory subunit 3 [Source:HGNC Symbol;Acc:HGNC:1173]			
84619	ZGPAT	zinc finger CCCH-type and G-patch domain containing [Source:HGNC Symbol;Acc:HGNC:15948]			
5688	PSMA7	proteasome 20S subunit alpha 7 [Source:HGNC Symbol;Acc:HGNC:9536]			
55209	SETD5	SET domain containing 5 [Source:HGNC Symbol;Acc:HGNC:25566]			
89122	TRIM4	tripartite motif containing 4 [Source:HGNC Symbol;Acc:HGNC:16275]			
5450	POU2AF1	POU class 2 homeobox associating factor 1 [Source:HGNC Symbol;Acc:HGNC:9211]			
3512	JCHAIN	joining chain of multimeric IgA and IgM [Source:HGNC Symbol;Acc:HGNC:5713]			
51237	MZB1	marginal zone B and B1 cell specific protein [Source:HGNC Symbol;Acc:HGNC:30125]			
3493	IGHA1	immunoglobulin heavy constant alpha 1 [Source:HGNC Symbol;Acc:HGNC:5478]			
27033	ZBTB32	zinc finger and BTB domain containing 32 [Source:HGNC Symbol;Acc:HGNC:16763]			
3688	ITGB1	integrin subunit beta 1 [Source:HGNC Symbol;Acc:HGNC:6153]			
3572	IL6ST	interleukin 6 signal transducer [Source:HGNC Symbol;Acc:HGNC:6021]			
57460	PPM1H	protein phosphatase, Mg2+/Mn2+ dependent 1H [Source:HGNC Symbol;Acc:HGNC:18583]			
115330	GPR146	G protein-coupled receptor 146 [Source:HGNC Symbol;Acc:HGNC:21718]			
6942	TCF20	transcription factor 20 [Source:HGNC Symbol;Acc:HGNC:11631]			
8728	ADAM19	ADAM metallopeptidase domain 19 [Source:HGNC Symbol;Acc:HGNC:197]			
115123	MARCHF3	membrane associated ring-CH-type finger 3 [Source:HGNC Symbol;Acc:HGNC:28728]			
2275	FHL3	four and a half LIM domains 3 [Source:HGNC Symbol;Acc:HGNC:3704]			

79188	TMEM43	transmembrane protein 43 [Source:HGNC Symbol;Acc:HGNC:28472]
10927	SPIN1	spindlin 1 [Source:HGNC Symbol;Acc:HGNC:11243]
58508	KMT2C	lysine methyltransferase 2C [Source:HGNC Symbol;Acc:HGNC:13726]
6415	SELENOW	selenoprotein W [Source:HGNC Symbol;Acc:HGNC:10752]
55824	PAG1	phosphoprotein membrane anchor with glycosphingolipid microdomains 1 [Source:HGNC Symbol;Acc:HGNC:30043]
55256	ADI1	acireductone dioxygenase 1 [Source:HGNC Symbol;Acc:HGNC:30576]
347733	TUBB2B	tubulin beta 2B class IIb [Source:HGNC Symbol;Acc:HGNC:30829]
867	CBL	Cbl proto-oncogene [Source:HGNC Symbol;Acc:HGNC:1541]
9537	TP53I11	tumor protein p53 inducible protein 11 [Source:HGNC Symbol;Acc:HGNC:16842]
220002	CYB561A3	cytochrome b561 family member A3 [Source:HGNC Symbol;Acc:HGNC:23014]
9600	PITPNM1	phosphatidylinositol transfer protein membrane associated 1 [Source:HGNC Symbol;Acc:HGNC:9003]
2161	F12	coagulation factor XII [Source:HGNC Symbol;Acc:HGNC:3530]
23531	MMD	monocyte to macrophage differentiation associated [Source:HGNC Symbol;Acc:HGNC:7153]
23215	PRRC2C	proline rich coiled-coil 2C [Source:HGNC Symbol;Acc:HGNC:24903]
10608	MXD4	MAX dimerization protein 4 [Source:HGNC Symbol;Acc:HGNC:13906]
55615	PRR5	proline rich 5 [Source:HGNC Symbol;Acc:HGNC:31682]
63893	UBE2O	ubiquitin conjugating enzyme E2 O [Source:HGNC Symbol;Acc:HGNC:29554]
11132	CAPN10	calpain 10 [Source:HGNC Symbol;Acc:HGNC:1477]
51199	NIN	ninein [Source:HGNC Symbol;Acc:HGNC:14906]
57580	PREX1	phosphatidylinositol-3,4,5-trisphosphate dependent Rac exchange factor 1 [Source:HGNC Symbol;Acc:HGNC:32594]
2035	EPB41	erythrocyte membrane protein band 4.1 [Source:HGNC Symbol;Acc:HGNC:3377]
7070	THY1	Thy-1 cell surface antigen [Source:HGNC Symbol;Acc:HGNC:11801]
8085	KMT2D	lysine methyltransferase 2D [Source:HGNC Symbol;Acc:HGNC:7133]
652968	CASTOR1	cytosolic arginine sensor for mTORC1 subunit 1 [Source:HGNC Symbol;Acc:HGNC:34423]
23683	PRKD3	protein kinase D3 [Source:HGNC Symbol;Acc:HGNC:9408]
5253	PHF2	PHD finger protein 2 [Source:HGNC Symbol;Acc:HGNC:8920]
65991	FUNDC2	FUN14 domain containing 2 [Source:HGNC Symbol;Acc:HGNC:24925]
5980	REV3L	REV3 like, DNA directed polymerase zeta catalytic subunit [Source:HGNC Symbol;Acc:HGNC:9968]
280636	SELENOH	selenoprotein H [Source:HGNC Symbol;Acc:HGNC:18251]
2022	ENG	endoglin [Source:HGNC Symbol;Acc:HGNC:3349]
51155	JPT1	Jupiter microtubule associated homolog 1 [Source:HGNC Symbol;Acc:HGNC:14569]
84649	DGAT2	diacylglycerol O-acyltransferase 2 [Source:HGNC Symbol;Acc:HGNC:16940]
79689	STEAP4	STEAP4 metalloreductase [Source:HGNC Symbol;Acc:HGNC:21923]
6302	TSPAN31	tetraspanin 31 [Source:HGNC Symbol;Acc:HGNC:10539]
10549	PRDX4	peroxiredoxin 4 [Source:HGNC Symbol;Acc:HGNC:17169]
65990	ANTKMT	adenine nucleotide translocase lysine methyltransferase [Source:HGNC Symbol;Acc:HGNC:14152]
8239	USP9X	ubiquitin specific peptidase 9 X-linked [Source:HGNC Symbol;Acc:HGNC:12632]
23275	POFUT2	protein O-fucosyltransferase 2 [Source:HGNC Symbol;Acc:HGNC:14683]
23144	ZC3H3	zinc finger CCCH-type containing 3 [Source:HGNC Symbol;Acc:HGNC:28972]
2971	GTF3A	general transcription factor IIIA [Source:HGNC Symbol;Acc:HGNC:4662]
598	BCL2L1	BCL2 like 1 [Source:HGNC Symbol;Acc:HGNC:992]
11145	PLAAT3	phospholipase A and acyltransferase 3 [Source:HGNC Symbol;Acc:HGNC:17825]
27090	ST6GALNA	ST6 N-acetylgalactosaminide alpha-2,6-sialyltransferase 4 [Source:HGNC Symbol;Acc:HGNC:17846]
23625	FAM89B	family with sequence similarity 89 member B [Source:HGNC Symbol;Acc:HGNC:16708]
81926	ABHD17A	abhydrolase domain containing 17A, depalmitoylase [Source:HGNC Symbol;Acc:HGNC:28756]
57492	ARID1B	AT-rich interaction domain 1B [Source:HGNC Symbol;Acc:HGNC:18040]
51129	ANGPTL4	angiopoietin like 4 [Source:HGNC Symbol;Acc:HGNC:16039]
348262	MCRIP1	MAPK regulated corepressor interacting protein 1 [Source:HGNC Symbol;Acc:HGNC:28007]
3315	HSPB1	heat shock protein family B (small) member 1 [Source:HGNC Symbol;Acc:HGNC:5246]
83416	FCRL5	Fc receptor like 5 [Source:HGNC Symbol;Acc:HGNC:18508]
9274	BCL7C	BAF chromatin remodeling complex subunit BCL7C [Source:HGNC Symbol;Acc:HGNC:1006]
8636	SSNA1	SS nuclear autoantigen 1 [Source:HGNC Symbol;Acc:HGNC:11321]
10430	TMEM147	transmembrane protein 147 [Source:HGNC Symbol;Acc:HGNC:30414]
113000	RPUSD1	RNA pseudouridine synthase domain containing 1 [Source:HGNC Symbol;Acc:HGNC:14173]
1487	CTBP1	C-terminal binding protein 1 [Source:HGNC Symbol;Acc:HGNC:2494]
122704	MRPL52	mitochondrial ribosomal protein L52 [Source:HGNC Symbol;Acc:HGNC:16655]
4728	NDUFS8	NADH:ubiquinone oxidoreductase core subunit S8 [Source:HGNC Symbol;Acc:HGNC:7715]
7494	XBP1	X-box binding protein 1 [Source:HGNC Symbol;Acc:HGNC:12801]
11190	CEP250	centrosomal protein 250 [Source:HGNC Symbol;Acc:HGNC:1859]
5434	POLR2E	RNA polymerase II, I and III subunit E [Source:HGNC Symbol;Acc:HGNC:9192]
90480	GADD45GI	GADD45G interacting protein 1 [Source:HGNC Symbol;Acc:HGNC:29996]
6182	MRPL12	mitochondrial ribosomal protein L12 [Source:HGNC Symbol;Acc:HGNC:10378]
1163	CKS1B	CDC28 protein kinase regulatory subunit 1B [Source:HGNC Symbol;Acc:HGNC:19083]
440275	EIF2AK4	eukaryotic translation initiation factor 2 alpha kinase 4 [Source:HGNC Symbol;Acc:HGNC:19687]
51006	SLC35C2	solute carrier family 35 member C2 [Source:HGNC Symbol;Acc:HGNC:17117]
58485	TRAPPC1	trafficking protein particle complex 1 [Source:HGNC Symbol;Acc:HGNC:19894]
4597	MVD	mevalonate diphosphate decarboxylase [Source:HGNC Symbol;Acc:HGNC:7529]
4519	MT-CYB	mitochondrially encoded cytochrome b [Source:HGNC Symbol;Acc:HGNC:7427]
55738	ARFGAP1	ADP ribosylation factor GTPase activating protein 1 [Source:HGNC Symbol;Acc:HGNC:15852]
2335	FN1	fibronectin 1 [Source:HGNC Symbol;Acc:HGNC:3778]
347862	GATD1	glutamine amidotransferase like class 1 domain containing 1 [Source:HGNC Symbol;Acc:HGNC:26616]

199990	FAAP20	FA core complex associated protein 20 [Source:HGNC Symbol;Acc:HGNC:26428]
65993	MRPS34	mitochondrial ribosomal protein S34 [Source:HGNC Symbol;Acc:HGNC:16618]
7776	ZNF236	zinc finger protein 236 [Source:HGNC Symbol;Acc:HGNC:13028]
2812	GP1BB	glycoprotein Ib platelet subunit beta [Source:HGNC Symbol;Acc:HGNC:4440]
7186	TRAF2	TNF receptor associated factor 2 [Source:HGNC Symbol;Acc:HGNC:12032]
3681	ITGAD	integrin subunit alpha D [Source:HGNC Symbol;Acc:HGNC:6146]
4052	LTBP1	latent transforming growth factor beta binding protein 1 [Source:HGNC Symbol;Acc:HGNC:6714]
4537	MT-ND3	mitochondrially encoded NADH:ubiquinone oxidoreductase core subunit 3 [Source:HGNC Symbol;Acc:HGNC:7458]
4536	MT-ND2	mitochondrially encoded NADH:ubiquinone oxidoreductase core subunit 2 [Source:HGNC Symbol;Acc:HGNC:7456]
4535	MT-ND1	mitochondrially encoded NADH:ubiquinone oxidoreductase core subunit 1 [Source:HGNC Symbol;Acc:HGNC:7455]
342618	SLFN14	schlafen family member 14 [Source:HGNC Symbol;Acc:HGNC:32689]
4508	MT-ATP6	mitochondrially encoded ATP synthase membrane subunit 6 [Source:HGNC Symbol;Acc:HGNC:7414]
4509	MT-ATP8	mitochondrially encoded ATP synthase membrane subunit 8 [Source:HGNC Symbol;Acc:HGNC:7415]
4514	MT-CO3	mitochondrially encoded cytochrome c oxidase III [Source:HGNC Symbol;Acc:HGNC:7422]
4538	MT-ND4	mitochondrially encoded NADH:ubiquinone oxidoreductase core subunit 4 [Source:HGNC Symbol;Acc:HGNC:7459]
4541	MT-ND6	mitochondrially encoded NADH:ubiquinone oxidoreductase core subunit 6 [Source:HGNC Symbol;Acc:HGNC:7462]

Table S8-Reads derived from 16S rDNA samples

sample-id	input	filtered	denoised	merged	non-chimeric	
#q2:types	numeric	numeric	numeric	numeric	numeric	
7224_2	125595	58288	58288	45131	26323	
7228R_7	351573	158344	158344	144667	109916	
7229_9	261559	123968	123968	116312	85594	
7230_12	400829	175386	175386	163508	127014	
7234_16	6197	2504	2504	1915	1665	
7257_2	123597	59591	59591	54918	45681	
7261_7	143735	54588	54588	51789	44113	
7262_9	163214	65310	65310	60865	46715	
7263_12	263979	110408	110408	98538	74561	
7267_16	394253	156412	156412	147797	116671	
7291_20	157563	35157	35157	32120	27224	
7296_33	3239	866	866	594	525	sample removed (low counts)
7297_30	323808	139174	139174	129573	101252	
7298_31	330371	144826	144826	118258	68292	
7303_20	437363	175164	175164	165287	121040	
7308_33	118921	52556	52556	48801	39242	
7309_30	343970	142145	142145	132420	108199	
7310_31	438046	180108	180108	160804	101975	
neg-PCR-FV1	1481	71	71	45	45	
neg-PCR-FV2	748	78	78	66	66	
2736_0	1510485	816390	816390	815991	749772	
2736-FV_0	237509	130121	130121	130114	120575	
6300-FV_0	880588	409058	409058	400121	243775	
6311_0	939454	448008	448008	446521	396246	
6311-10_0	635707	300003	300003	299258	277369	
6311-FV_0	140797	67451	67451	67312	60390	
